# Dietary and lifestyle factors for primary prevention of nephrolithiasis: a systematic review and meta-analysis

**DOI:** 10.1186/s12882-020-01925-3

**Published:** 2020-07-11

**Authors:** Bing-Biao Lin, Ming-En Lin, Rong-Hua Huang, Ying-Kai Hong, Bing-Liang Lin, Xue-Jun He

**Affiliations:** 1 Department of Urology, The First Affiliated Hospital of Shantou University Medical College, 57 Changping Road, Shantou, Guangdong China; 2 Shantou University Medical College, 22 Xinling Road, Shantou, Guangdong China; 3 Department of Anesthesiology, The First Affiliated Hospital of Shantou University Medical College, 57 Changping Road, Shantou, Guangdong China; 4 School of Public Health, Guangdong Medical University, No.1 City Avenue Songshan Lake Sci. & Tech. Industry Park, Dongguan, Guangdong China

**Keywords:** Nephrolithiasis, Diet, Obesity, Epidemiology, Systematic review, Meta-analysis

## Abstract

**Background:**

Dietary and lifestyle factors may play an important role in the increasing prevalence of nephrolithiasis. We aimed to review and quantify the associations between lifestyle factors and incident nephrolithiasis and suggest lifestyle changes for the primary prevention of nephrolithiasis.

**Methods:**

PubMed, EMBASE, and Cochrane Library were searched up to May 2019, for observational studies and randomized controlled trials (RCTs) that assessed modifiable lifestyle factors and risk of nephrolithiasis in adults. Pooled relative risks (RRs) and 95% confidence intervals (CIs) were computed using a random effects model. The I^2^ statistic was employed to evaluate heterogeneity. Subgroup analysis, sensitivity analysis and meta-regression were also conducted whenever possible.

**Results:**

Fifty relevant articles with 1,322,133 participants and 21,030 cases in total were identified. Prominent risk factors for incident stones were body mass index (1.39,1.27–1.52), dietary sodium (1.38, 1.21–1.56), fructose, meat, animal protein, and soda. In contrast, protective factors included fluid intake (0.55, 0.51–0.60), a Dietary Approaches to Stop Hypertension (DASH) style diet (0.69, 0.64–0.75), alcohol (0.69, 0.56–0.85), water, coffee, tea, vegetables, fruits, dietary fiber, dietary calcium (0.83, 0.76–0.90), and potassium. Vitamin D (1.22, 1.01–1.49) and calcium (1.16, 1.00–1.35) supplementation alone increased the risk of stones in meta-analyses of observational studies, but not in RCTs, where the cosupplementation conferred significant risk.

**Conclusions:**

Several modifiable factors, notably fluid intake, dietary patterns, and obesity, were significantly associated with nephrolithiasis. Long-term RCTs are required to investigate the cost-effectiveness of dietary patterns for stone prevention. The independent and combined effects of vitamin D and calcium supplementation on nephrolithiasis need further elucidation.

## Background

Nephrolithiasis, though usually manifested as a benign painful condition, poses a tremendous threat to the health care system and individual well-being. It incurs an enormous cost estimated at US $2.1 billion in 2000 and with a projected annual increase of US $1.24 billion by 2030 as a result of population growth and the pandemics of diabetes and obesity [1, 2]. It has been recognized as a systemic disease in recent decades given its connections with multiple diseases [3, 4]. Nephrolithiasis increases the risk of bone fractures, chronic kidney disease, renal cell carcinoma, cardiovascular diseases and mortality [5–10]. Worse quality of life was also observed among stone formers, especially non-White individuals with lower income [11].

Nevertheless, the prevalence and incidence of kidney stones have continued to grow globally, and despite the higher prevalence in males, up to 10.6% in 2007–2010, the gender gap appears to be closing due to a disproportionally increased prevalence in females [12–14]. Although increased accidental detection by imaging studies is contributed to the growing prevalence, lifestyle and dietary factors have also been suggested to play an important role [15]. This was evidenced by one cohort study [16] showing that more than half of incident stones were attributable to lifestyle factors. To reduce stone formation and socioeconomic burden, preventive strategies targeting modifiable lifestyle factors may be effective for people at high risk of recurrent stones and even incident ones [17]. However, epidemiologic studies and meta-analyses that assessed the associations between kidney stones and different lifestyle factors have demonstrated inconsistent results [18–23]. Although a recent meta-analysis reported a negative association with fluid consumption, its methodology was less robust in that it included cross-sectional studies and duplicated cohorts and combined data from both recurrent and incident stone formers [21].

We, therefore, conducted a comprehensive systematic review and meta-analysis with an aim to build an evidence-based list of modifiable lifestyle and dietary factors and to establish corresponding lifestyle recommendations.

## Methods

Two authors (BBL and MEL) independently searched the databases, screened the articles retrieved, abstracted the data and assessed the quality of included studies. Discrepancies were resolved by consensus. This study followed MOOSE (Meta-analysis of Observational Studies in Epidemiology) and PRISMA (Preferred Reporting Items for Systematic Reviews and Meta-Analyses) guidelines.

### Search strategy and study selection

PubMed, EMBASE and Cochrane Library were searched from inception to May 2019 using the terms “risk” AND “kidney stone” with limitation to publications in English. Full search terms and included studies are listed in Additional file 1.

Publications were considered eligible if they fulfilled the following inclusion criteria: i) observational studies or RCTs of adults without prior history of nephrolithiasis; ii) assessment of at least one modifiable risk factor; and iii) provided risk estimates with 95% CI, or enough information to calculate them. Exclusion criteria were also applied: i) population with recurrent or prevalent kidney stones as this condition might have affected their dietary behaviors; ii) studies featuring nonmodifiable risk factors, genetic or urinary lithogenic factors; and iii) cross-sectional studies or RCTs with short duration of follow-up (e.g., ≤1 year) and small sample size (< 100 participants). Bibliographies of included studies and related reviews were hand-searched for additional publications. If multiple articles reported on the same cohorts, we only selected the most recent one unless different risk factors were assessed. When a single paper reported both individual and pooled risk estimates of different cohorts, or presented gender-specific results, we chose to analyze them individually in the meta-analysis.

### Data abstraction and quality assessment

We recorded the first author’s last name, publication year, country, study design, study period, participants’ genders and baseline ages, the number of participants and cases, assessment factors and methods, exposure comparison, outcomes, maximally adjusted risk estimates with 95% CIs, and confounding factors by using piloted data extraction sheets. The incidence rate ratios (IRRs) and 95% CIs were inverted for one article [16] that used the beneficial groups as reference. Quality was assessed using the Newcastle Ottawa Scale (NOS) for observational studies (Additional file 2) and using the Cochrane risk of bias tool for RCTs (Additional file 3).

### Statistical analysis

Observational studies and RCTs were analyzed separately. Heterogeneous exposure categorization existed across most observational studies, which rendered exposure standardization impossible. As a result, we generated pooled RRs by comparing the highest and lowest exposure levels. In addition, a random effect model was used to counter variations in study design and population. Heterogeneity was quantitatively assessed by the I^2^ statistic, with values greater than 50 and 75% representing moderate and substantial heterogeneity, respectively. To identify sources of heterogeneity, subgroup and meta-regression analyses were performed according to study design, gender, region, study quality, number of cases, durations of follow-up and confounding factors whenever applicable. We used funnel plot, Egger’s test and the trim-and-fill method to evaluate publication bias and small study effects only if the number of included studies was ≥10. Sensitivity analyses were performed to evaluate each study’s effect on the overall risk estimates (Additional file 4). Statistical analyses were performed by STATA 13.1 (College Station, TX, USA). All *P* values were two-sided with *P* < 0.05 regarded as statistically significant.

## Results

**Fig. 1:**
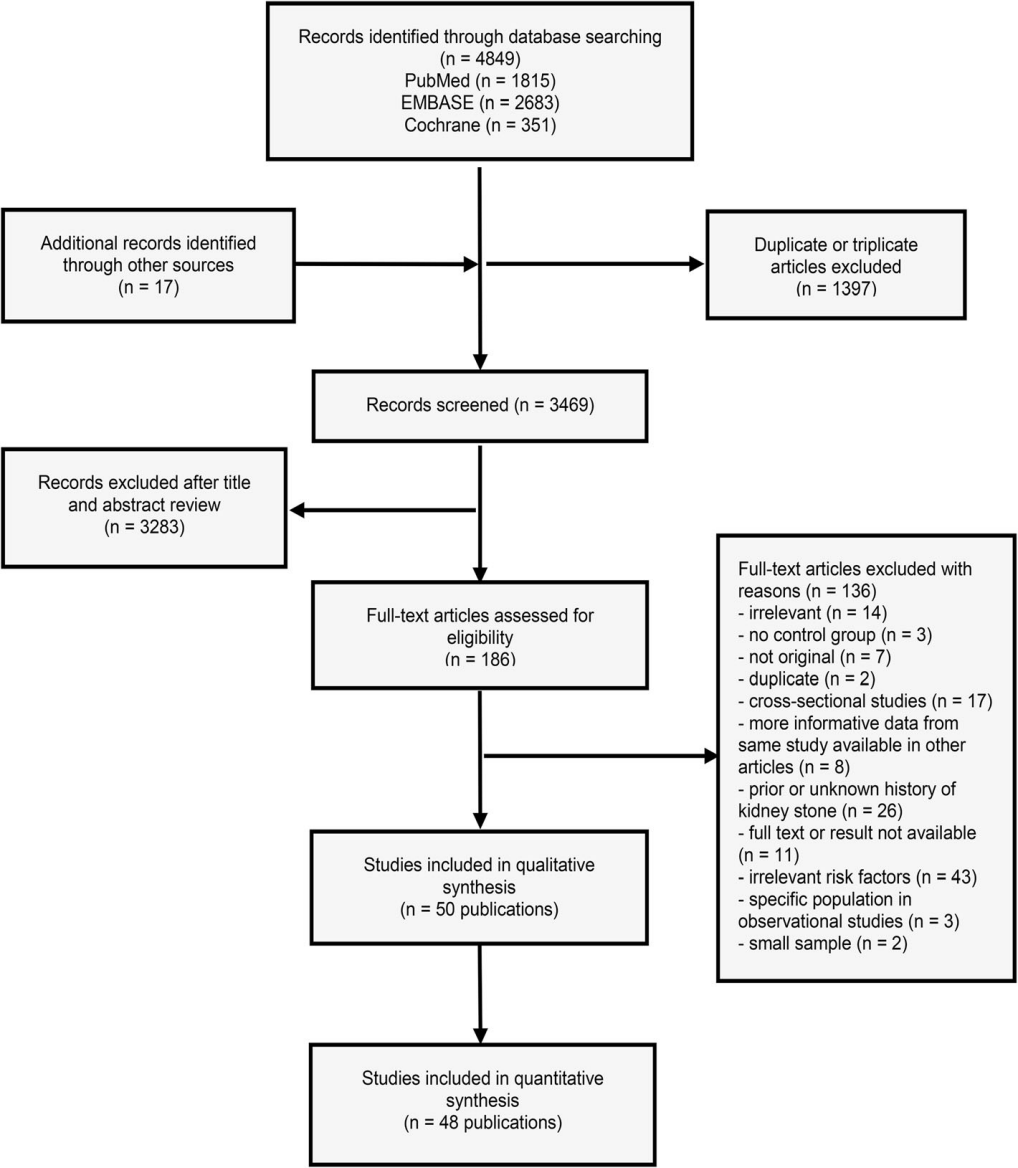
Flow chart of the study selection process

The selection process with exclusion reasons is presented in Fig. 1. The literature search yielded 4849 articles, of which 186 were potentially eligible after title and abstract screening. Through full-text review, 50 articles containing a total of 1,322,133 participants and 21,030 cases with study duration ranging from 1 year to 26 years were included (Additional file 5). Of 50 publications, 34 were cohort studies (33 prospective and 1 retrospective),12 were RCTs, and 4 were case-control studies. The included studies either used self-report, abdominal ultrasound, medical record review or International Classification Disease code to ascertain a diagnosis of nephrolithiasis. Some articles contained 2–3 study populations and assessed multiple risk factors, which encompassed obesity (*n* = 15), beverage (*n* = 12), diet (*n* = 19), physical activity (*n* = 4), energy intake (*n* = 3), vitamins and calcium supplements (*n* = 22). All the included RCTs reported data available for secondary analysis of the associations with vitamin D and calcium supplementation.

### Diet

Dietary factors were assessed in 10 observational studies (19 publications), of which 8 were cohort studies conducted within the USA and Europe and 2 were case-controlled studies in the USA and China.

**Fig. 2:**
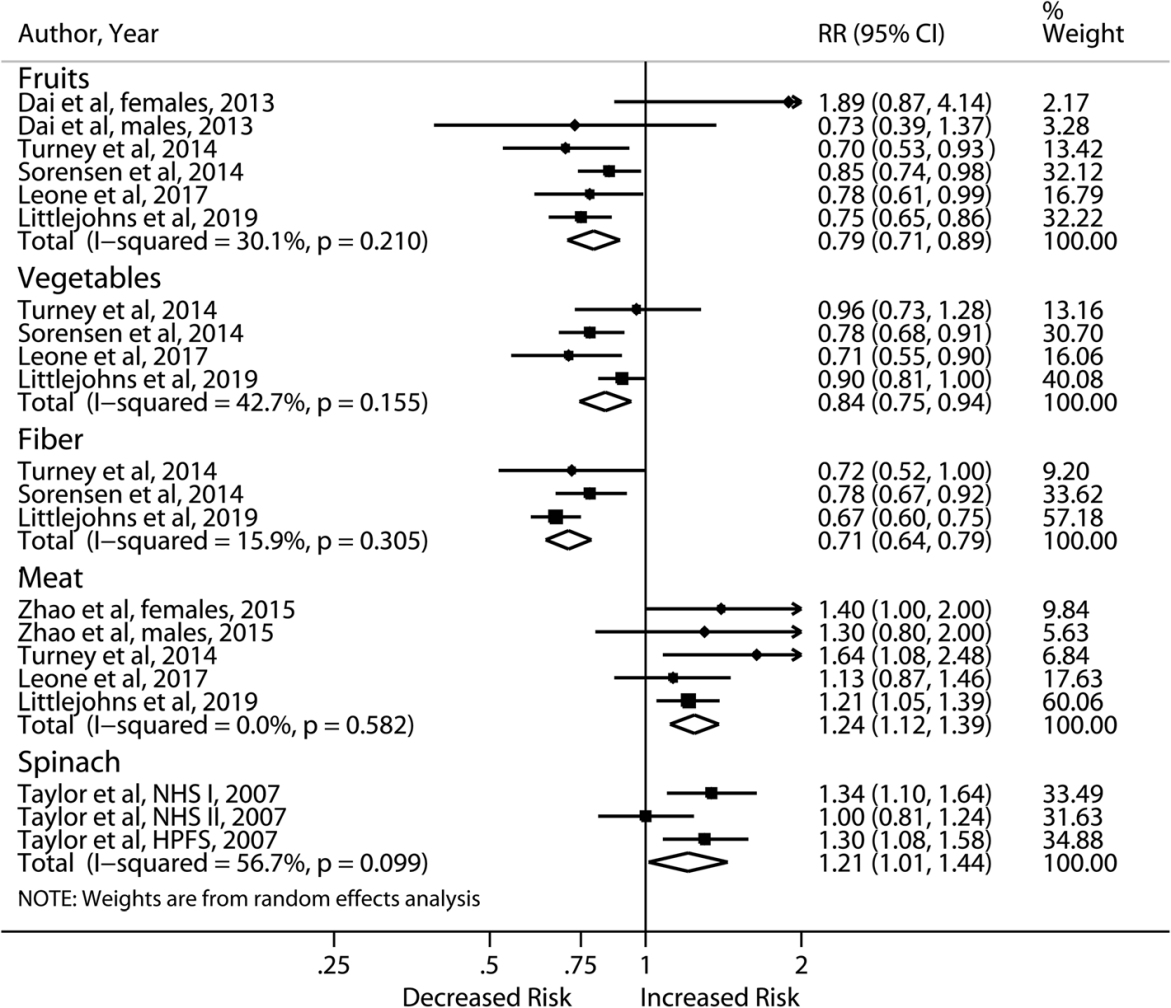
Forest plot of meta-analysis comparing highest vs lowest level of dietary intakes and risk of incident nephrolithiasis

In the meta-analysis of high vs low intake (Fig. 2), a high intake of meat significantly increased the risk of kidney stones (RR: 1.24; 95% CI, 1.12–1.39). No significant heterogeneity was present and sensitivity analysis produced consistent results. Two studies further investigated red meat, processed meat and poultry with inconsistent results [24, 25]. Both reported a 20–53% higher risk of stones for high intake of red meat and null association for poultry consumption. Only one showed significantly increased risk with processed (RR: 1.34; 95% CI, 1.12–1.59) and poultry meat consumption (RR: 1.22; 95% CI, 1.00–1.49) [24]. There was no association between fish intake and incident stones in 2 studies [24, 26]. Five cohort studies reported on animal protein, with only one study [27] showing the opposite direction of risk estimate without adjustment for body mass index (BMI) and diabetes [27–29]. A meta-analysis of the other 4 yielded borderline significant risk (RR: 1.1; 95% CI, 1.02–1.19) with no heterogeneity.

As shown in Fig. 2, risk of kidney stones was markedly reduced by high intake of fruit (RR: 0.79; 95% CI, 0.71–0.89), dietary fiber (RR: 0.71; 95% CI, 0.64–0.79) and vegetables (RR: 0.84; 95% CI, 0.75–0.94). Of note, the significance of both vegetables and dietary fiber would be lost and heterogeneity became significant if we included 2 studies [27, 30] that demonstrated opposite results with higher intake of vegetables and fiber respectively. These 2 studies were excluded as one [30] claimed the most common vegetables taken was spinach, which increased the risk in the meta-analysis of the three cohorts [31] (RR: 1.21; 95% CI, 1.01–1.44), and another [27] did not adjust for BMI and diabetes. The risk of nephrolithiasis observed in high spinach intake could be partly explained by the high oxalate content (RR: 1.16; 95% CI, 1.05–1.29). No significant heterogeneity was present.

**Fig. 3:**
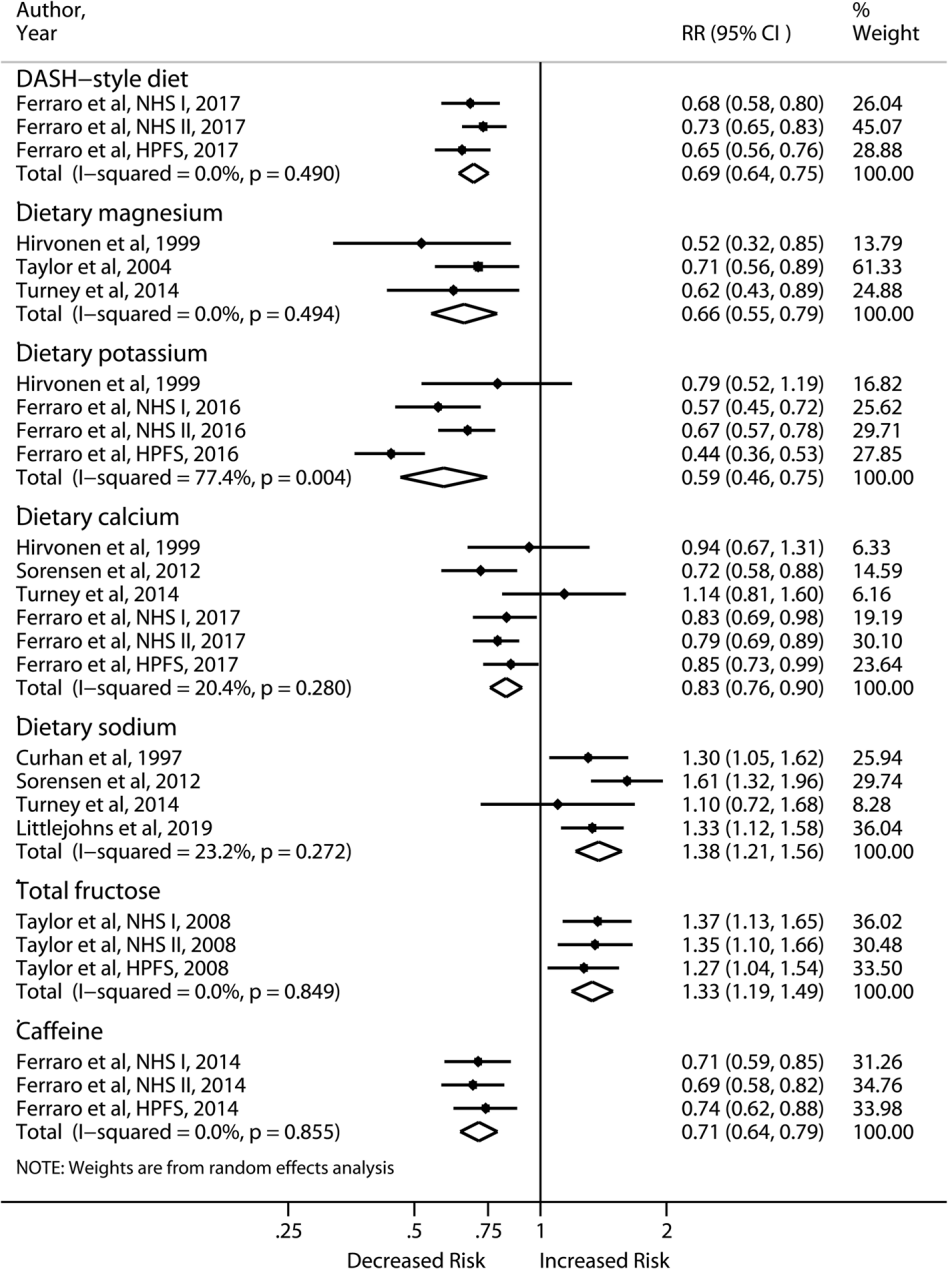
Forest plot of meta-analysis comparing the highest vs lowest level of dietary intakes and risk of incident nephrolithiasis

Dietary calcium (RR: 0.83; 95% CI, 0.76–0.90), potassium (RR: 0.59; 95% CI, 0.46–0.75) and magnesium (RR: 0.66; 95% CI, 0.55–0.79) intake were inversely associated with the risk of incident nephrolithiasis, whereas high intake of dietary sodium increased the risk by 38% (Fig. 3). Only the meta-analysis of dietary potassium produced substantial heterogeneity (I^2^ = 77.4%). The pooled result of 3 cohorts [16] for the DASH style diet revealed significant risk reduction (RR: 0.69; 95% CI, 0.64–0.75). A similar association with the Mediterranean dietary pattern was reported by Leon et al. (HR: 0.64; 95% CI, 0.48–0.87) [26]. Sensitivity analyses were all consistent across studies.

### Beverage

Thirteen studies (12 publications) including 11 prospective cohort studies and 2 case-controlled studies investigated the effect of beverages on kidney stones, of which 5 were conducted within the USA, 4 in Europe, and 4 in Asia.

**Fig. 4:**
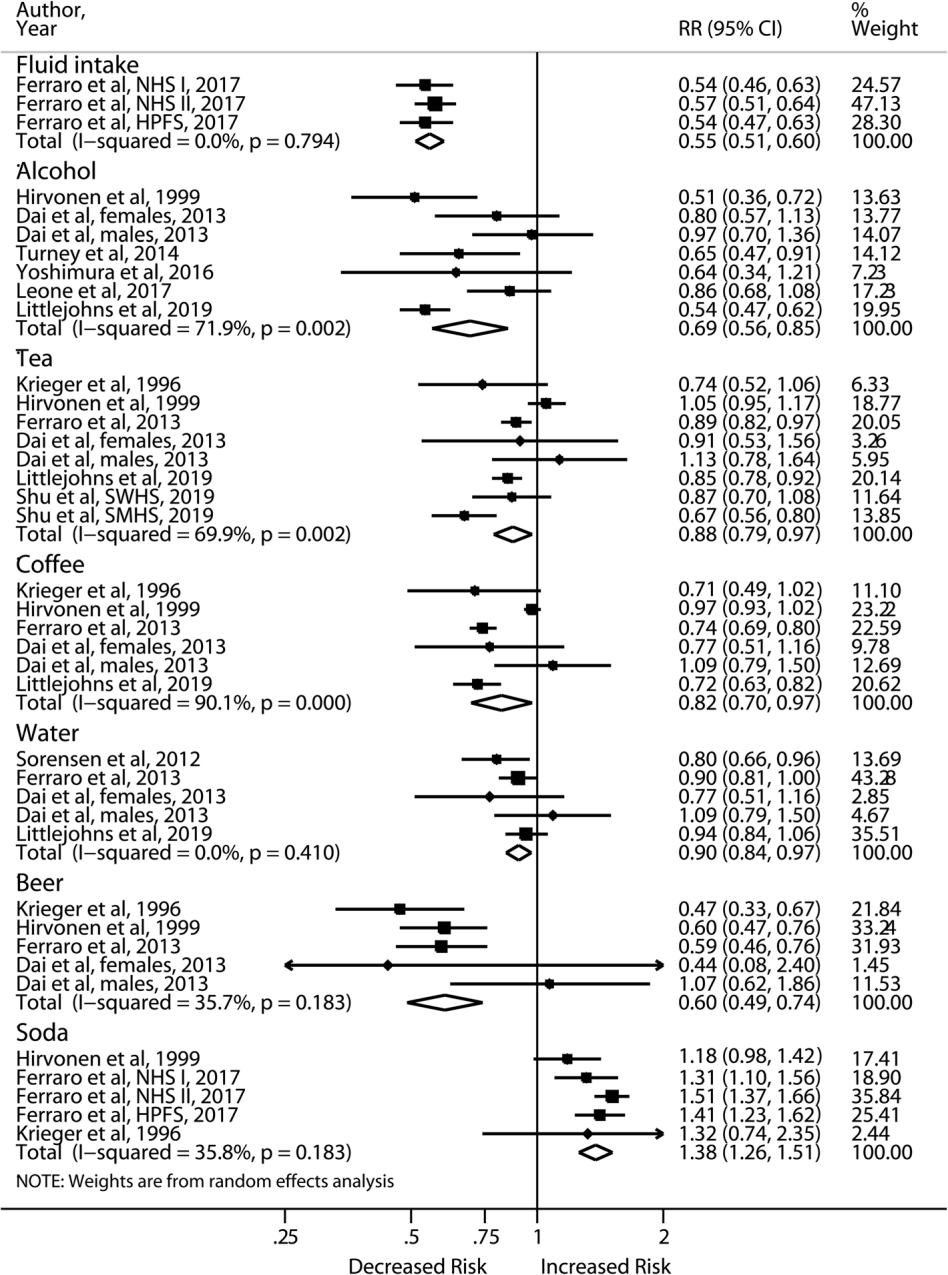
Forest plot of meta-analysis comparing the highest vs lowest level of beverages and risk of incident nephrolithiasis

Total fluid intake up to 2 l/d reduced the risk of incident stones by almost half (RR: 0.56; 95% CI, 0.48–0.65) when compared with less than 1 l/d (Fig. 4). Substantial heterogeneity (I^2^ = 79.3%) was reduced to I^2^ = 0.0%, while the association remained significant (RR: 0.55; 95% CI, 0.51–0.60) after excluding 2 studies without adjustment for dietary factors [24, 27]. Increased alcohol and beer consumption were associated with 31 and 40% lower risk of incident stones, respectively. Several studies further investigated different types of alcoholic beverages. Ferraro et al. [32] reported significant pooled RRs of 0.69, 0.67 and 0.88 for red wine, white wine and liquor, respectively, in three cohort studies, whereas 2 studies showed nonsignificant results [27, 33]. Lower risks were found for individuals consuming high vs low amounts of coffee (RR: 0.82; 95% CI, 0.7–0.97), tea (RR: 0.88; 95% CI, 0.79–0.97), and water (RR: 0.90; 95% CI, 0.84–0.97), as demonstrated in Fig. 4. Increased intake of caffeine, found in tea and coffee, also reduced the risk by 29% [34]. The meta-analysis of high vs low consumption of soda revealed an increased risk of incident stones (RR: 1.38; 95% CI, 1.26–1.51) with low heterogeneity (I^2^ = 35.8%) after excluding one study [33] that was not controlled for dietary factors. This observation might be partly attributable to fructose content (RR: 1.33; 95% CI, 1.19–1.49). Regarding various juices, whole and skim milk, no significant associations were found in 2 articles [27, 32]. Sensitivity analyses yielded similar results for various beverages.

### Vitamins and calcium supplementation

**Fig. 5:**
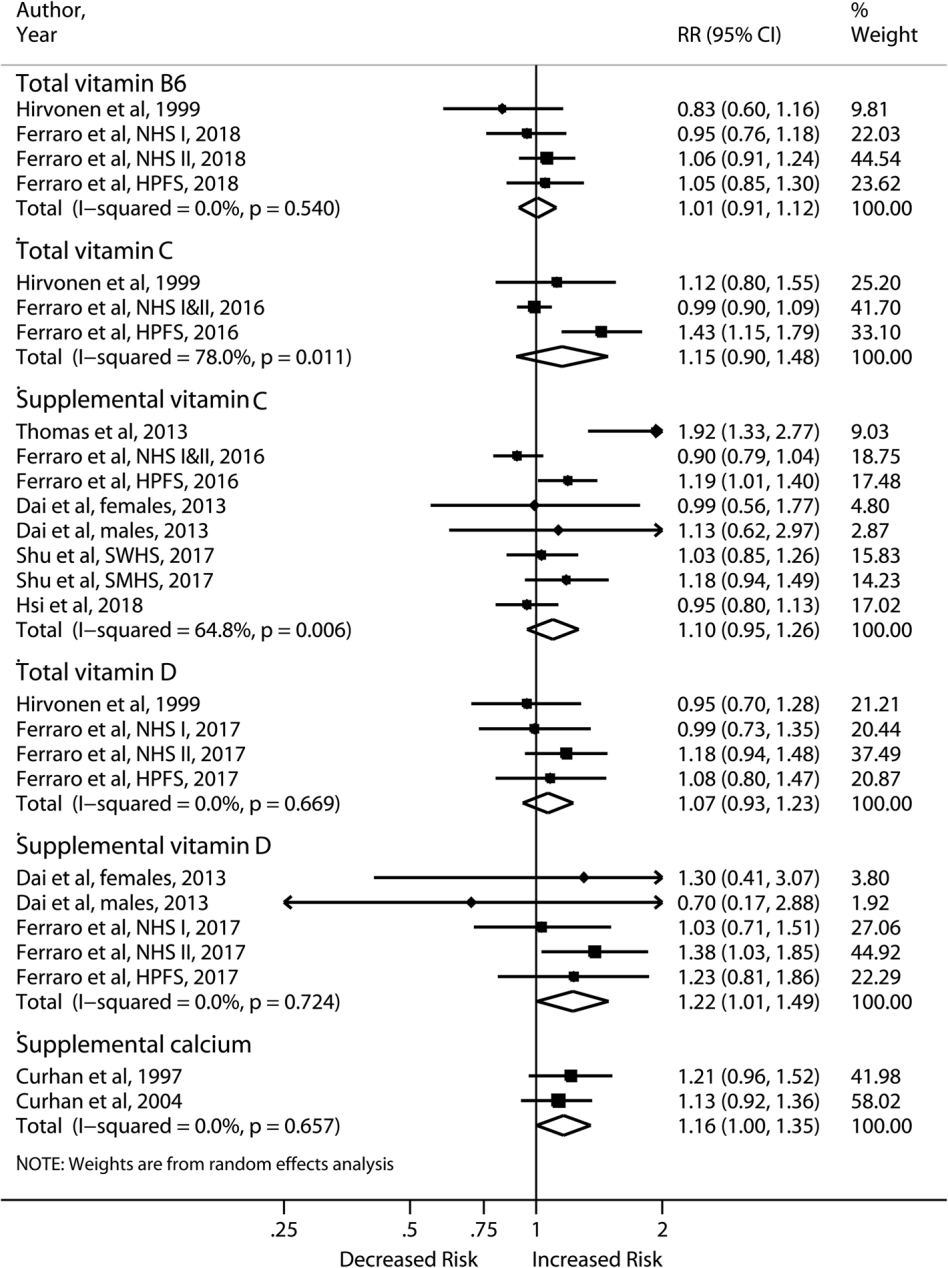
Forest plot of highest vs lowest category of vitamin and calcium supplementation and risk of incident nephrolithiasis in observational studies

**Fig. 6:**
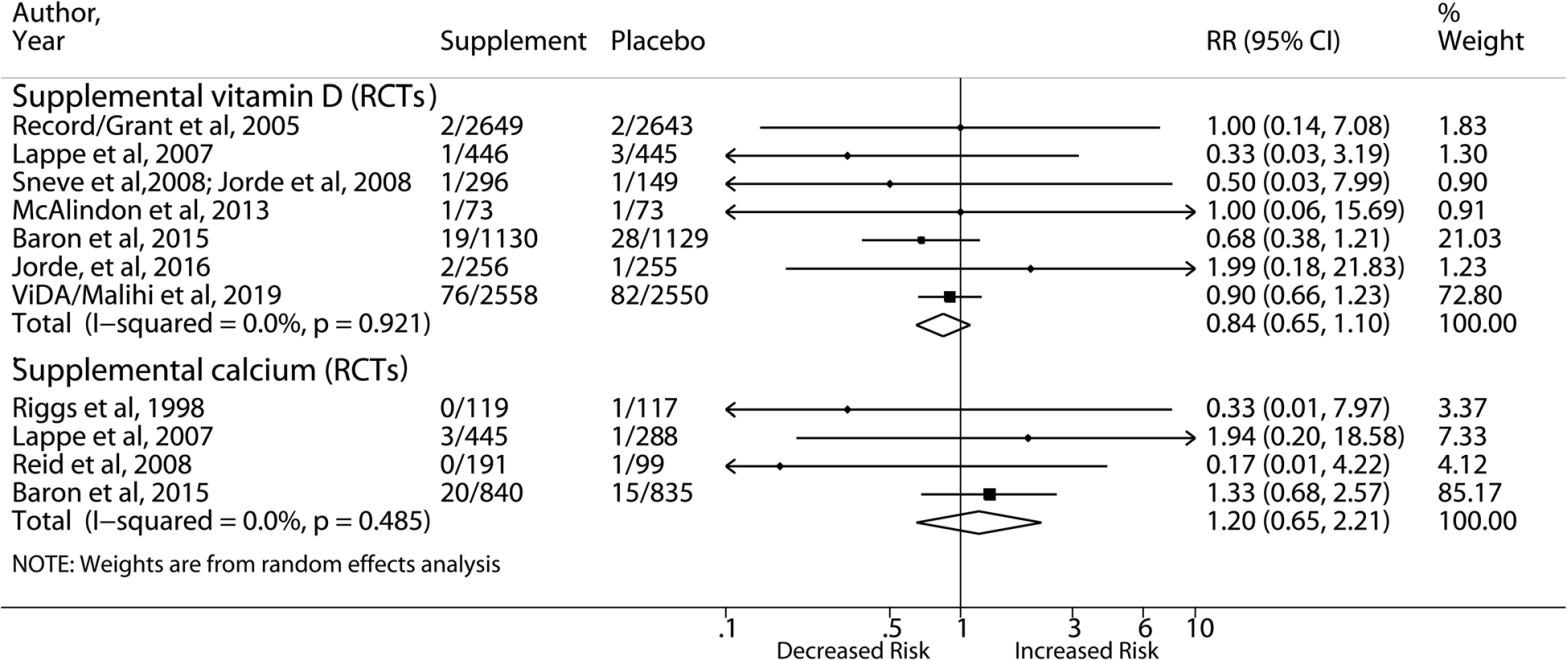
Forest plot of highest vs lowest category of vitamin and calcium supplementation and risk of incident nephrolithiasis in RCTs. RECORD, Randomized Evaluation of Calcium or Vitamin D; ViDA, Vitamin D Assessment; RCTs, randomized controlled trials; RR, relative risk; CI, confidence interval

Ten observational studies and 11 RCTs (22 publications) assessed the association with vitamin consumption and calcium supplement. The meta-analyses of observational studies and RCTs are summarized in Fig. 5 and Fig. 6, respectively.

No significant associations with incident stones were observed for high intake of dietary vitamin B6 and total vitamin D. Meta-analyses of total and supplemental vitamin C showed nonsignificantly increased risks of incident stones with moderate and substantial heterogeneity, respectively. The pooled results of 2 female cohorts [35, 36] for supplemental calcium > 501 mg/d yielded a significant RR of 1.16 (95% CI, 1.00–1.35). In contrast, 4 RCTs of supplemental calcium (600–1600 mg/d) produced a null association without heterogeneity. Supplemental vitamin D increased the risk of kidney stones in 5 observational studies (RR: 1.22; 95% CI, 1.01–1.49) but not in 7 RCTs (RR: 0.84; 95% CI, 0.65–1.10). The VITAL study was the largest RCT in terms of sample size and cases but it did not exclude participants with a history of kidney stones. When we included the VITAL study, the pooled RR of RCTs changed from 0.84 to 1.06 (95% CI, 0.95–1.19). Sensitivity analyses demonstrated similar results. The meta-analysis of vitamin D plus calcium supplementation in 3 RCTs was no different from the previous meta-analysis by Kahwati et al. [37], showing that the cosupplementation was a significant stone predictor (RR: 1.18; 95% CI, 1.04–1.35) (Data not shown in the figure). The result was driven by the Women’s Health Initiative RCT (RR: 1.17; 95% CI,1.03–1.34) [38].

### Obesity

Seventeen studies in 15 articles examined the effect of obesity on incident kidney stones, with 16, 5 and 2 studies investigating BMI, waist circumference (WC) and waist-to-hip ratio (WHR), respectively. Of 17 studies, 7 were conducted in the Americas, 8 in Asia, and 2 in Europe.

**Fig. 7:**
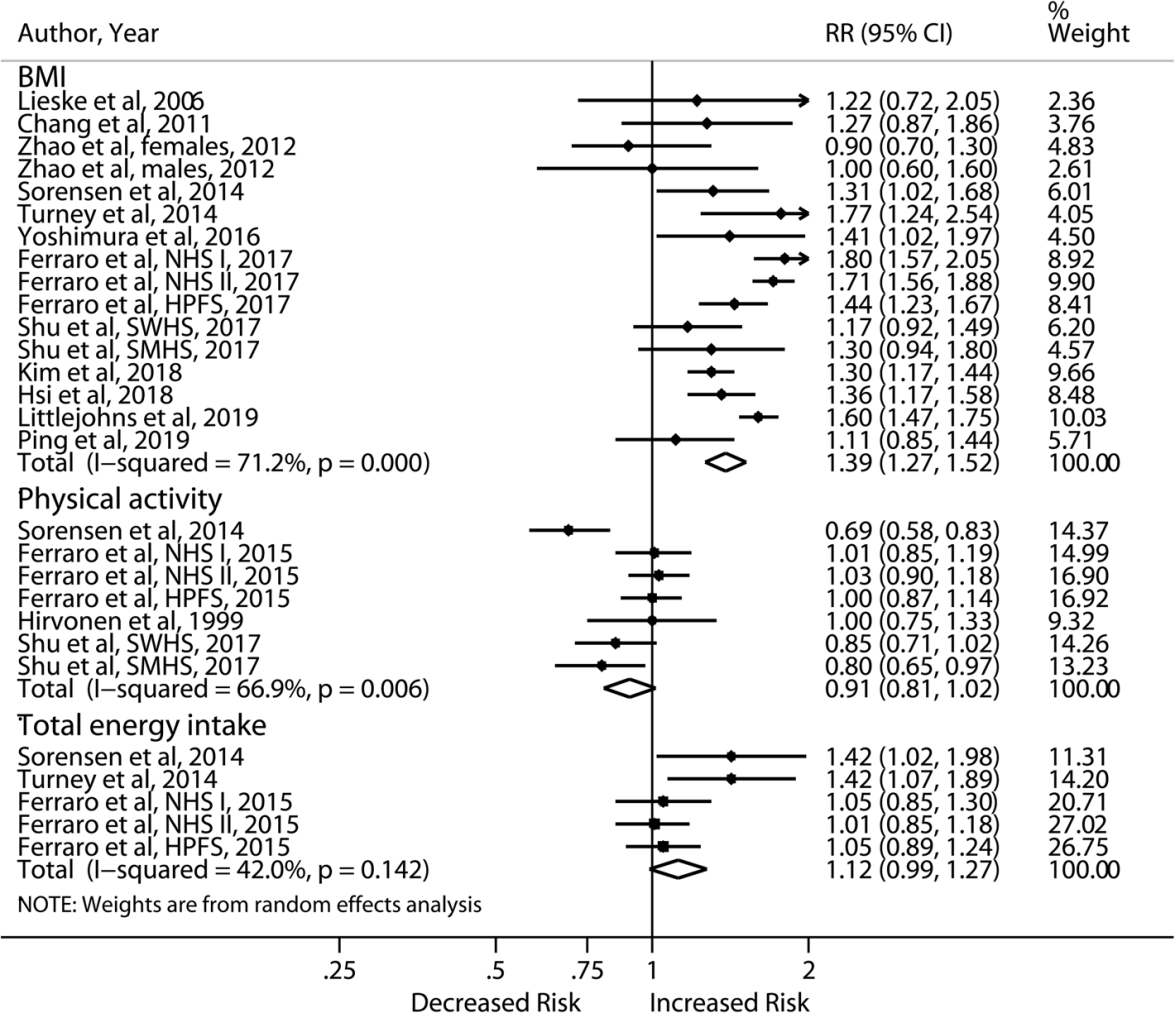
Forest plot of highest vs lowest category of BMI, physical activity, energy intake and risk of incident nephrolithiasis. BMI, body mass index; NHS, Nurses’ Health Study; HPFS, Health Professional Follow-up Study; SWHS, Shanghai Women’s Health Study; SMHS, Shanghai Men’s Health Study; RR, relative risk; CI, confidence interval

By comparing the highest with the lowest level of BMI (Fig. 7), we observed a significant 39% increase in the risk of first stones, although moderate heterogeneity (I^2^ = 71.2%) and publication bias (*P* = 0.002) were present (Additional file 6). According to subgroup analyses (Additional file 7), the effect of BMI was comparable for both genders but appeared to be stronger in the Americas (RR: 1.53; 95% CI, 1.36–1.71) and Europe (RR: 1.61; 95% CI, 1.48–1.75). Meta-regression revealed that region explained 61.84% of the between-study variance in addition to thiazide use, fluid intake, duration of follow-up, study quality and assessment methods. Sensitivity analysis showed stable results and no new study was added with Duval’s trim and fill method.

One study [39] reported the risk of kidney stones per 1 unit increase in BMI and was therefore not included in the analysis. Two studies showed significantly increased risk in males with high WHR and inconsistent results for females [30, 40].

### Physical activity

Physical activity was assessed in 4 articles with 7 cohort studies. Four studies were conducted in the USA, 2 in China, and 1 in Finland. The meta-analysis of the 7 studies revealed slightly but not significantly decreased risk of incident stones (RR: 0.91; 95% CI, 0.81–1.02) with increased physical activity level (Fig. 7). Sensitivity analysis yielded similar results, and moderate heterogeneity (I^2^ = 71.2%) existed.

### Energy intake

Five cohort studies (3 publications) investigated the association between energy intake and nephrolithiasis. A nonsignificant 12% increase in the risk of incident stones was found with energy intake ≥2500 kcal/d (RR: 1.12; 95% CI, 0.99–1.27) (Fig. 7). The result of sensitivity analysis remained nonsignificant except when the Nurses’ Health Study (NHS) II [41] was excluded.

## Discussion

To our knowledge, this is the first systematic review and meta-analysis to collectively investigate a wide range of modifiable lifestyle factors and to clarify their strength of association with incident kidney stones by comparing the highest vs lowest categories of exposures. The findings suggested that total fluid intake, water, coffee, tea, alcohol, beer, fruit, vegetables, dietary fiber, dietary potassium, magnesium, and calcium decreased the risk of kidney stones, whereas high BMI, total meat intake, animal protein, dietary sodium, spinach, oxalate, fructose and soda increased the risk. Total and supplemental vitamin C intakes were linked with nonsignificant risk of renal stones. Supplemental vitamin D and calcium alone increased the risk in observational studies but not in RCTs. In contrast, cosupplementation conferred the risk in RCTs. No significant associations were detected with physical activity, energy intake, dietary vitamin B6 and total vitamin D.

### Diet

Increased consumption of total meat and animal protein conferred a significant risk of nephrolithiasis. Ferraro et al. collected and assessed the 24-h urine samples, showing that higher intake of animal protein was associated with 54 (95% CI, 30–78) mg/d lower urine citrate, 24 (95% CI, 13–35) mg/d higher urine uric acid, 0.13 (95% CI, 0.09–0.17) lower urine pH, and higher relative supersaturation for uric acid [28]. This, in turn, favors the development of calcium and uric acid stone formation [42, 43]. Despite convincing proof of animal protein as a significant stone predictor, epidemiologic and experimental studies were rather sparse for specific sources of animal protein. A study in 2014 examined the effects of fish, chicken, beef intake on 24-h urine metabolites among healthy subjects [44]. Although they all linked to an increased tendency to stone formation, beef consumption posed a slightly higher risk for calcium oxalate stone development. In contrast to the null association with fish intake observed in 2 cohort studies, this comparative metabolic study suggested a positive association between fish and higher urinary uric acid level. The adverse effect of animal protein could be counterbalanced by increased dietary potassium, which had 39 (95% CI, 13–65) mg/d higher urine citrate, 0.15 (95% CI, 0.10–0.19) higher urine pH, and significantly lower relative supersaturations for calcium oxalate and uric acid when compared with lower intake of potassium [28, 45]. Fruits, vegetables and dietary fiber are important sources of phytate, which reduces the risk of stones by inhibiting urinary crystallization of calcium salts [46]. Despite their effect to promote urinary oxalate excretion, fruits and vegetables have been shown to inhibit stone development by increasing urinary volume, urine pH, and urinary excretion of potassium, magnesium and citrate [47]. A diet rich in fiber, vegetables, and fruits and reduced in animal protein and total meat is therefore recommended.

An inverse association of dietary calcium with kidney stones was found because high dietary calcium prevented oxalate absorption by forming calcium oxalate complex in the guts, thus reducing urinary oxalate excretion [48]. However, calcium consumption that exceeded the amount for complexing exogenous oxalate potentially elevated urinary calcium concentration and the risk of stones [49]. Increased dietary sodium conferred a 38% higher risk of stones because of its positive association with hypercalciuria [50]. Taken together, a low-salt diet with a dietary calcium uptake of 1000–1200 mg/day is favored to reduce the risk of calcium-containing stones.

A DASH-style diet, which contains a large number of fruits, vegetables and dairy products and low saturated fat [51], significantly lowered the risk of kidney stones by 31% in the meta-analysis. It also reduced the risk of cardiovascular diseases [51]. In a 24-h urine analysis, adherence to the DASH diet corresponded to higher urinary magnesium, potassium, citrate, pH, and lower urine supersaturations for calcium oxalate (women only) and uric acid [52]. Advice on dietary patterns instead of isolated nutrients is more feasible because the meals we consume consist of various nutrients in different proportions, and the overall effect rely on the interactions between each dietary factor. This could be evidenced by the opposite effect of calcium taken with a meal or solitarily on the risk of nephrolithiasis. To date, few RCTs have been conducted to testify to the efficacy of dietary patterns for stone prevention. Borghi, et al. demonstrated that a diet of normal calcium with low animal protein and salt was superior to a low-calcium diet for the prevention of recurrent stones among hypercalciuric patients [53]. Another RCT showed that a DASH-style diet nonsignificantly decreased urine calcium oxalate supersaturation among stone formers with hyperoxaluria when compared with an oxalate-restricted diet [54]. Large-scale RCTs are required to investigate the cost-effectiveness of various dietary patterns for stone prevention.

### Beverages

In our meta-analyses, increased fluid, alcohol and beer uptake decreased the risk of nephrolithiasis, followed by high intake of coffee, tea and water. In contrast, a high intake of soda promoted stone formation. One RCT demonstrated that urinary volume could be manipulated to reduce the recurrence rate by high water intake [55]. It is more sensible to titrate fluid uptake to obtain urine output > 2 l/d, as the goal of increasing fluid intake is to increase urine output and concurrently lower urine supersaturation. Another meta-analysis of beverage was in line with most of our findings except for soda, which it identified as insignificant [20]. The inconsistency could be explained by our inclusion of the latest studies that demonstrated opposite results for soda use after a longer follow-up. Alcohol could promote urine formation by inducing diuresis and inhibiting the secretion of anti-diuretic hormones [56]. Nevertheless, alcohol should be discouraged given its detrimental effects on human health. Both coffee and tea elicited diuretic and natriuretic responses via caffeine despite their effect in increasing urine calcium excretion [34, 57, 58]. In the 24-h urinary analysis, a higher intake of caffeine was associated with significantly lower urinary supersaturation for both uric acid and calcium oxalate [34]. High intake of soda elevated urinary excretion of calcium, uric acid and oxalate [59, 60].

### Vitamin and calcium supplementation

Calcium and vitamin D supplementation was controversial due to inconsistent meta-analyses of RCTs and observational studies. The NHS I [35] reported significantly increased risk among elderly women while others did not [36, 61]. The author attributed the risk to not taking supplemental calcium with meals in that the beneficial effect of dietary calcium on oxalate could not be replicated under such circumstances. This hypothesis was supported by another study [62] showing significantly lower urinary oxalate when calcium was taken with a meal instead of at bedtime. Regarding supplemental vitamin D, this updated meta-analysis of RCTs concurred with 2 previous meta-analyses [22, 37] but contradicted that by Bjelakovic et al. [19], who included RCTs comparing supplemental vitamin D and calcium with placebo. The discrepancy could be partly explained by the possible synergistic effect of cosupplementation, which was supported by the Women Health Initiative trials [38]. In addition, an animal study showed the highest urinary calcium excretion and significant kidney calcification among rats with cosupplementation [63]. Nevertheless, additional studies are needed to elucidate their associations with kidney stones.

### Obesity

Pooled analysis of high vs low BMI yielded a significant 39% increased risk of incident stones. Insulin resistance, a feature of obesity [64] and type 2 diabetes, promoted uric acid stone formation by impairing renal ammoniagenesis and decreasing urine pH [65]. Furthermore, obesity was connected with increased levels of urinary sodium, uric acid, phosphate and oxalate [66, 67]. Considering that different exposure categorization may account for the stronger risk observed in western countries, we then limited data analysis to cohort studies that compared BMI ≥ 30 kg/m2 with < 25 kg/m2. The result also showed a greater magnitude of risk in the Americas (RR: 1.54; 95% CI, 1.37–1.74) than in Asia (RR: 1.26; 95% CI, 1.16–1.37), indicating that obesity contributed to the higher prevalence in western countries.

Primary preventive measures, if well developed and implemented among the stone prone population, may limit the substantial negative impact of nephrolithiasis on the health care system and individuals. It was shown that primary preventive measures such as increased water intake could significantly decrease cost and stone burden in virtual cohorts [17]. As the pathophysiological features of kidney stone formation are believed to remain the same regardless of a history of nephrolithiasis, it is likely that the results in the meta-analyses also apply to subjects with former nephrolithiasis. In line with this assumption, current guidelines used mostly observational studies for incident kidney stones to make dietary suggestions for secondary prevention [68, 69]. To endorse preventive measures, a higher level of evidence, such as RCTs, are needed to confirm the cost-effectiveness of dietary patterns for stone prevention. Currently, it is less likely and less motivating for people to change diets for the sole purpose of preventing incident stones. Therefore, greater priority should be given to evaluate the cost-effectiveness of dietary patterns for the prevention of stone recurrence in RCTs. Our study mainly underscores the important role of lifestyle factors in stone development and serves to promote education, awareness, and lifestyle changes.

There are various limitations to our study. First, although a few articles reported that calcium-oxalate stones were the predominant type in small subsamples of NHS I (77%), NHS II (79%) and HPFS (86%) [41], most studies included lacked information on stone composition, rendering it impossible to appreciate the strength of associations with specific types of stones. Second, most studies did not have biochemical data such as urinary compositions, which would be useful to explain the biological effect of specific dietary factors on stone formation. Third, although we used maximally adjusted risk estimates whenever provided, residual confounding by unknown or unmeasured factors could not be excluded. Fourth, whether the impact of lifestyle factors on stone risk varies from gender remained unknown due to a paucity of included studies. Another pitfall of the meta-analysis includes the failure to perform dose-response analysis owing to various reference and exposure groups and insufficient data. In addition, most RCTs were not powered for nephrolithiasis due to small sample sizes, short follow-up and low event rates. The participants in the included RCTs were mostly above 50 years old. Therefore, the findings of RCT meta-analyses are most directly generalizable to those above 50 years of age. Care should be taken when attempting to extend the results across age groups.

## Conclusion

The summarized evidence in this study indicated that several lifestyle factors, such as BMI, beverages and dietary patterns, were significantly associated with the risk of nephrolithiasis. Adherence to specific dietary patterns is likely to have a far-reaching effect in reducing the stone burden. However, long-term RCTs are needed to confirm the cost-effectiveness of dietary patterns for the reduction of the incidence and recurrence rates of kidney stones. Future studies on vitamin D and calcium supplementation could help clarify their respective and combined effects on kidney stone formation.

## Supplementary information

**Additional file 1.** Search strategies of PubMed, EMBASE, and Cochrane Library and the resulting list of included studies.

**Additional file 2.** Summary of Newcastle Ottawa Scale scores for observational studies.

**Additional file 3.** Risk of bias summary and graph for included randomized controlled trials in meta-analysis.

**Additional file 4.** Sensitivity analyses of the included studies.

**Additional file 5.** Characteristics of the included studies of incident kidney stones.

**Additional file 6.** Funnel plot (A) and Egger’s test (B) for publication bias and small study effects among studies assessing the association between body mass index and risk of incident nephrolithiasis.

**Additional file 7.** Subgroup meta-analyses and meta-regression of body mass index and incident kidney stones.

## Data Availability

All data generated or analyzed during this study are included in this article.

## Abbreviations

RCTs: Randomized controlled trials
WHR: Waist-to-hip ratio;
DASH: Dietary Approaches to Stop Hypertension

## References

[1] Antonelli JA, Maalouf NM, Pearle MS, Lotan Y (2014). Use of the national health and nutrition examination survey to calculate the impact of obesity and diabetes on cost and prevalence of urolithiasis in 2030. Eur Urol.

[2] Ziemba JB, Matlaga BR (2017). Epidemiology and economics of nephrolithiasis. Invest Clin Urol.

[3] Taylor EN, Stampfer MJ, Curhan GC (2005). Diabetes mellitus and the risk of nephrolithiasis. Kidney Int.

[4] Kim SY, Song CM, Lim H, Lim MS, Bang W, Choi HG (2019). Bidirectional association between gallstones and renal stones: two longitudinal follow-up studies using a national sample cohort. Sci Rep.

[5] Alexander RT, Hemmelgarn BR, Wiebe N, Bello A, Samuel S, Klarenbach SW (2014). Kidney stones and cardiovascular events: a cohort study. Clin J Am Soc Nephrol.

[6] van de Pol JAA, van den Brandt PA, Schouten LJ (2019). Kidney stones and the risk of renal cell carcinoma and upper tract urothelial carcinoma: the Netherlands cohort study. Br J Cancer.

[7] Ferraro PM, Taylor EN, Eisner BH, Gambaro G, Rimm EB, Mukamal KJ (2013). History of kidney stones and the risk of coronary heart disease. JAMA.

[8] Dhondup T, Kittanamongkolchai W, Vaughan LE, Mehta RA, Chhina JK, Enders FT (2018). Risk of ESRD and mortality in kidney and bladder stone formers. Am J Kidney Dis.

[9] Sakhaee K (2008). Nephrolithiasis as a systemic disorder. Curr Opin Nephrol Hypertens.

[10] Taylor EN, Feskanich D, Paik JM, Curhan GC (2016). Nephrolithiasis and risk of incident bone fracture. J Urol.

[11] Ahmad TR, Tzou DT, Usawachintachit M, Reliford-Titus S, Wu C, Goodman J (2019). Low income and nonwhite race are strongly associated with worse quality of life in patients with nephrolithiasis. J Urol.

[12] Romero V, Akpinar H, Assimos DG (2010). Kidney stones: a global picture of prevalence, incidence, and associated risk factors. Rev Urol.

[13] Scales CD, Curtis LH, Norris RD, Springhart WP, Sur RL, Schulman KA (2007). Changing gender prevalence of stone disease. J Urol.

[14] Scales CD, Smith AC, Hanley JM, Saigal CS (2012). Prevalence of kidney stones in the United States. Eur Urol.

[15] Straub M, Hautmann RE (2005). Developments in stone prevention. Curr Opin Urol.

[16] Ferraro PM, Taylor EN, Gambaro G, Curhan GC (2017). Dietary and lifestyle risk factors associated with incident kidney stones in men and women. J Urol.

[17] Lotan Y, Buendia Jimenez I, Lenoir-Wijnkoop I, Daudon M, Molinier L, Tack I (2012). Primary prevention of nephrolithiasis is cost-effective for a national healthcare system. BJU Int.

[18] Fink HA, Akornor JW, Garimella PS, MacDonald R, Cutting A, Rutks IR (2009). Diet, fluid, or supplements for secondary prevention of nephrolithiasis: a systematic review and meta-analysis of randomized trials. Eur Urol.

[19] Bjelakovic G, Gluud LL, Nikolova D, Whitfield K, Krstic G, Wetterslev J (2014). Vitamin D supplementation for prevention of cancer in adults. Cochrane Database Syst Rev.

[20] Xu C, Zhang C, Wang XL, Liu TZ, Zeng XT, Li S (2015). Self-fluid Management in Prevention of kidney stones: a PRISMA-compliant systematic review and dose-response meta-analysis of observational studies. Medicine (Baltimore).

[21] Cheungpasitporn W, Rossetti S, Friend K, Erickson SB, Lieske JC (2016). Treatment effect, adherence, and safety of high fluid intake for the prevention of incident and recurrent kidney stones: a systematic review and meta-analysis. J Nephrol.

[22] Malihi Z, Wu Z, Stewart AW, Lawes CM, Scragg R (2016). Hypercalcemia, hypercalciuria, and kidney stones in long-term studies of vitamin D supplementation: a systematic review and meta-analysis. Am J Clin Nutr.

[23] Aune D, Mahamat-Saleh Y, Norat T, Riboli E (2018). Body fatness, diabetes, physical activity and risk of kidney stones: a systematic review and meta-analysis of cohort studies. Eur J Epidemiol.

[24] Littlejohns TJ, Neal NL, Bradbury KE, Heers H, Allen NE, Turney BW. Fluid intake and dietary factors and the risk of incident kidney stones in UK biobank: a population-based prospective cohort study. Eur Urol Focus. 2019. 10.1016/j.euf.2019.05.002. 10.1016/j.euf.2019.05.002 31085062

[25] Turney BW, Appleby PN, Reynard JM, Noble JG, Key TJ, Allen NE (2014). Diet and risk of kidney stones in the Oxford cohort of the European prospective investigation into cancer and nutrition (EPIC). Eur J Epidemiol.

[26] Leone A, Fernandez-Montero A, de la Fuente-Arrillaga C, Martinez-Gonzalez MA, Bertoli S, Battezzati A (2017). Adherence to the Mediterranean dietary pattern and incidence of nephrolithiasis in the Seguimiento Universidad de Navarra follow-up (SUN) cohort. Am J Kidney Dis.

[27] Hirvonen T, Pietinen P, Virtanen M, Albanes D, Virtamo J (1999). Nutrient intake and use of beverages and the risk of kidney stones among male smokers. Am J Epidemiol.

[28] Ferraro PM, Mandel EI, Curhan GC, Gambaro G, Taylor EN (2016). Dietary protein and potassium, diet-dependent net acid load, and risk of incident kidney stones. Clin J Am Soc Nephrol.

[29] Sorensen MD, Kahn AJ, Reiner AP, Tseng TY, Shikany JM, Wallace RB (2012). Impact of nutritional factors on incident kidney stone formation: a report from the WHI OS. J Urol.

[30] Zhao A, Dai M, Chen YJ, Chang HE, Liu AP, Wang PY (2015). Risk factors associated with nephrolithiasis: a case-control study in China. Asia-Pac J Public Health.

[31] Taylor EN, Curhan GC (2007). Oxalate intake and the risk for nephrolithiasis. J Am Soc Nephrol.

[32] Ferraro PM, Taylor EN, Gambaro G, Curhan GC (2013). Soda and other beverages and the risk of kidney stones. Clin J Am Soc Nephrol.

[33] Dai M, Zhao A, Liu A, You L, Wang P (2013). Dietary factors and risk of kidney stone: a case-control study in southern China. J Ren Nutr.

[34] Ferraro PM, Taylor EN, Gambaro G, Curhan GC (2014). Caffeine intake and the risk of kidney stones. Am J Clin Nutr.

[35] Curhan GC, Willett WC, Speizer FE, Spiegelman D, Stampfer MJ (1997). Comparison of dietary calcium with supplemental calcium and other nutrients as factors affecting the risk for kidney stones in women. Ann Intern Med.

[36] Curhan GC, Willett WC, Knight EL, Stampfer MJ (2004). Dietary factors and the risk of incident kidney stones in younger women: Nurses’ health study II. Arch Intern Med.

[37] Kahwati LC, Weber RP, Pan H, Gourlay M, LeBlanc E, Coker-Schwimmer M (2018). Vitamin D, calcium, or combined supplementation for the primary prevention of fractures in community-dwelling adults: evidence report and systematic review for the US preventive services task force. Jama.

[38] Wallace RB, Wactawski-Wende J, O'Sullivan MJ, Larson JC, Cochrane B, Gass M (2011). Urinary tract stone occurrence in the Women's Health Initiative (WHI) randomized clinical trial of calcium and vitamin D supplements. Am J Clin Nutr.

[39] Oda E (2014). Overweight and high-sensitivity C-reactive protein are weakly associated with kidney stone formation in Japanese men. Int J Urol.

[40] Shu X, Cai H, Xiang YB, Li H, Lipworth L, Miller NL (2017). Nephrolithiasis among middle aged and elderly urban Chinese: a report from prospective cohort studies in Shanghai. J Endourol.

[41] Ferraro PM, Curhan GC, Sorensen MD, Gambaro G, Taylor EN (2015). Physical activity, energy intake and the risk of incident kidney stones. J Urol.

[42] Breslau NA, Brinkley L, Hill KD, Pak CY (1988). Relationship of animal protein-rich diet to kidney stone formation and calcium metabolism. J Clin Endocrinol Metab.

[43] Kok DJ, Iestra JA, Doorenbos CJ, Papapoulos SE (1990). The effects of dietary excesses in animal protein and in sodium on the composition and the crystallization kinetics of calcium oxalate monohydrate in urines of healthy men. J Clin Endocrinol Metab.

[44] Tracy CR, Best S, Bagrodia A, Poindexter JR, Adams-Huet B, Sakhaee K (2014). Animal protein and the risk of kidney stones: a comparative metabolic study of animal protein sources. J Urol.

[45] Lemann J, Pleuss JA, Gray RW, Hoffmann RG (1991). Potassium administration reduces and potassium deprivation increases urinary calcium excretion in healthy adults [corrected]. Kidney Int.

[46] Grases F (2007). Phytate acts as an inhibitor in formation of renal calculi. Front Biosci.

[47] Meschi T, Maggiore U, Fiaccadori E, Schianchi T, Bosi S, Adorni G (2004). The effect of fruits and vegetables on urinary stone risk factors. Kidney Int.

[48] Lemann J, Pleuss JA, Worcester EM, Hornick L, Schrab D, Hoffmann RG (1996). Urinary oxalate excretion increases with body size and decreases with increasing dietary calcium intake among healthy adults. Kidney Int.

[49] Matsumoto ED, Heller HJ, Adams-Huet B, Brinkley LJ, Pak CYC, Pearle MS (2006). Effect of high and low calcium diets on stone forming risk during Liberal oxalate intake. J Urol.

[50] Massey LK, Whiting SJ (1995). Dietary salt, urinary calcium, and kidney stone risk. Nutr Rev.

[51] Eckel RH, Jakicic JM, Ard JD, de Jesus JM, Miller NH, Hubbard VS (2014). 2013 AHA/ACC guideline on lifestyle management to reduce cardiovascular risk. Circulation.

[52] Taylor EN, Stampfer MJ, Mount DB, Curhan GC (2010). DASH-style diet and 24-hour urine composition. Clin J Am Soc Nephrol.

[53] Borghi L, Schianchi T, Meschi T, Guerra A, Allegri F, Maggiore U (2002). Comparison of two diets for the prevention of recurrent stones in idiopathic hypercalciuria. N Engl J Med.

[54] Noori N, Honarkar E, Goldfarb DS, Kalantar-Zadeh K, Taheri M, Shakhssalim N (2014). Urinary lithogenic risk profile in recurrent stone formers with hyperoxaluria: a randomized controlled trial comparing DASH (dietary approaches to stop hypertension)-style and low-oxalate diets. Am J Kidney Dis.

[55] Borghi L, Meschi T, Amato F, Briganti A, Novarini A, Giannini A (1996). Urinary volume, water and recurrences in idiopathic calcium nephrolithiasis: a 5-year randomized prospective study. J Urol.

[56] Eisenhofer G, Johnson RH (1982). Effect of ethanol ingestion on plasma vasopressin and water balance in humans. Am J Phys.

[57] Rieg T, Steigele H, Schnermann J, Richter K, Osswald H, Vallon V (2005). Requirement of intact adenosine A1 receptors for the diuretic and natriuretic action of the methylxanthines theophylline and caffeine. J Pharmacol Exp Ther.

[58] Peerapen P, Thongboonkerd V (2018). Caffeine in kidney stone disease: risk or benefit?. Adv Nutr.

[59] Nguyen NU, Dumoulin G, Henriet MT, Regnard J (1995). Increase in urinary calcium and oxalate after fructose infusion. Horm Metab Res.

[60] Fox IH, Kelley WN (1972). Studies on the mechanism of fructose-induced hyperuricemia in man. Metab Clin Exp.

[61] Taylor EN, Stampfer MJ, Curhan GC (2004). Dietary factors and the risk of incident kidney stones in men: new insights after 14 years of follow-up. J Am Soc Nephrol.

[62] Domrongkitchaiporn S, Sopassathit W, Stitchantrakul W, Prapaipanich S, Ingsathit A, Rajatanavin R (2004). Schedule of taking calcium supplement and the risk of nephrolithiasis. Kidney Int.

[63] Letavernier E, Verrier C, Goussard F, Perez J, Huguet L, Haymann JP (2016). Calcium and vitamin D have a synergistic role in a rat model of kidney stone disease. Kidney Int.

[64] Ronti T, Lupattelli G, Mannarino E (2006). The endocrine function of adipose tissue: an update. Clin Endocrinol.

[65] Daudon M, Traxer O, Conort P, Lacour B, Jungers P (2006). Type 2 diabetes increases the risk for uric acid stones. J Am Soc Nephrol.

[66] Esperto F, Marangella M, Miano R, Trinchieri A (2017). 24-hour urine parameters and body mass index in a large cohort of high risk renal stone formers patients. Eur Urol Suppl.

[67] Wollin DA, Skolarikos A, Preminger GM (2017). Obesity and metabolic stone disease. Curr Opin Urol.

[68] Dion M, Ankawi G, Chew B, Paterson R, Sultan N, Hoddinott P (2016). CUA guideline on the evaluation and medical management of the kidney stone patient - 2016 update. Can Urol Assoc J.

[69] Ziemba JB, Matlaga BR (2015). Guideline of guidelines: kidney stones. BJU Int.

