# Supplementary material for: Dietary and lifestyle factors for primary prevention of nephrolithiasis: a systematic review and meta-analysis
Source: BMC Nephrol. 2020 Jul 11;21:267. doi: 10.1186/s12882-020-01925-3 (PMC7353736; doi:10.1186/s12882-020-01925-3)
Supplement: Supplementary file 1 — Additional file 1. Search strategies of PubMed, EMBASE, and Cochrane Library and the resulting list of included studies. [file 12882_2020_1925_MOESM1_ESM.docx]

**Additional file 1:** **Search strategies of PubMed, EMBASE, and Cochrane Library and the resulting list of included studies.**

- **The search for each database was conducted from inception to May 2019. In general, we combined “kidney stone*” with “risk*” and applied filters for English, adults,case-control studies, cohort studies, and randomized controlled trials.**

1. **PubMed: Search ((((Risk[MeSH Terms]) OR risk*[Title/Abstract])) AND (((Nephrolithiasis[MeSH Terms]) OR Kidney Calculi[MeSH Terms]) OR ((((((((((((((((Calculi, Kidney[Title/Abstract]) OR Calculus, Kidney[Title/Abstract]) OR Kidney Calculus[Title/Abstract]) OR Nephrolith[Title/Abstract]) OR Renal Calculus[Title/Abstract]) OR Kidney Stones[Title/Abstract]) OR Kidney Stone[Title/Abstract]) OR Stone, Kidney[Title/Abstract]) OR Stones, Kidney[Title/Abstract]) OR Renal Calculi[Title/Abstract]) OR Calculi, Renal[Title/Abstract]) OR Calculus, Renal[Title/Abstract]) OR Renal Stones[Title/Abstract]) OR Renal Stone[Title/Abstract]) OR Stone, Renal[Title/Abstract]) OR Stones, Renal[Title/Abstract]))) AND ((((randomized controlled trial[pt] OR controlled clinical trial[pt] OR randomized[tiab] OR placebo[tiab] OR clinical trials as topic[mesh:noexp] OR randomly[tiab] OR trial[ti] NOT (animals[mh] NOT humans [mh])))) OR (("cohort studies"[mesh] OR "case-control studies"[mesh] OR "comparative study"[pt] OR "risk factors"[mesh] OR "cohort"[tw] OR "compared"[tw] OR "groups"[tw] OR "case control"[tw] OR "multivariate"[tw]))) Filters: Humans; English; Adult: 19+ years**
2. **Embase:**

**#20 #11 AND #14 AND #17 AND ([adult]/lim OR [aged]/lim) AND [english]/lim**

**#19 #11 AND #14 AND #17 AND ([adult]/lim OR [aged]/lim)**

**#18 #11 AND #14 AND #17**

**#17 #15 OR #16**

**#16 'clinical article'/exp OR 'controlled study'/exp OR 'major clinical study'/exp OR 'prospective study'/exp OR 'cohort analysis'/exp OR 'cohort':ti,ab OR 'compared':ti,ab OR 'groups':ti,ab OR 'case control':ti,ab OR 'multivariate':ti,ab 13086386**

**#15 'crossover procedure':de OR 'double-blind procedure':de OR 'randomized controlled trial':de OR 'single-blind procedure':de OR random*:de,ab,ti OR factorial*:de,ab,ti OR crossover*:de,ab,ti OR ((cross NEXT/1 over*):de,ab,ti) OR placebo*:de,ab,ti OR ((doubl* NEAR/1 blind*):de,ab,ti) OR ((singl* NEAR/1 blind*):de,ab,ti) OR assign*:de,ab,ti OR allocat*:de,ab,ti OR volunteer*:de,ab,ti**

**#14 #12 OR #13**

**#13 'risk*':ab,ti**

**#12 'risk'/exp**

**#11 #1 OR #2 OR #10**

**#10 #8 AND #9**

**#9 #5 OR #6 OR #7**

**#8 #3 OR #4**

**#7 'calculi':ab,ti**

**#6 'calculus':ab,ti**

**#5 'stone*':ab,ti**

**#4 'kidney':ab,ti**

**#3 'renal':ab,ti**

**#2 'nephrolith':ab,ti**

**#1 'nephrolithiasis'/exp**

1. **Cochrane Library:**

**#1 MeSH descriptor: [Kidney Calculi] explode all trees**

**#2 MeSH descriptor: [Nephrolithiasis] explode all trees**

**#3 (kidney):ti,ab,kw**

**#4 (renal):ti,ab,kw**

**#5 (stone*):ti,ab,kw**

**#6 (calculus):ti,ab,kw**

**#7 (calculi):ti,ab,kw**

**#8 #3 or #4**

**#9 #5 or #6 or #7**

**#10 #8 and #9**

**#11 (nephrolith):ti,ab,kw**

**#12 #1 or #2 or #10 or #11**

**#13 MeSH descriptor: [Risk] explode all trees**

**#14 (risk*):ti,ab,kw**

**#15 #13 or #14**

**#16 #12 and #15 in Trials**

- **List of studies included in systemic review and meta-analysis of lifestyle risk factors and incident kidney stones:**

(1-50)

1. Krieger JN, Kronmal RA, Coxon V, Wortley P, Thompson L, Sherrard DJ. Dietary and behavioral risk factors for urolithiasis: potential implications for prevention. American Journal of Kidney Diseases. 1996;28(2):195-201.

2. Curhan GC, Willett WC, Speizer FE, Spiegelman D, Stampfer MJ. Comparison of dietary calcium with supplemental calcium and other nutrients as factors affecting the risk for kidney stones in women. Annals of internal medicine. 1997;126(7):497-504.

3. Riggs BL, O'Fallon WM, Muhs J, O'Connor MK, Kumar R, Melton LJ. Long-Term Effects of Calcium Supplementation on Serum Parathyroid Hormone Level, Bone Turnover, and Bone Loss in Elderly Women. Journal of Bone and Mineral Research. 1998;13(2):168-74.

4. Hirvonen T, Pietinen P, Virtanen M, Albanes D, Virtamo J. Nutrient intake and use of beverages and the risk of kidney stones among male smokers. Am J Epidemiol. 1999;150(2):187-94.

5. Curhan GC, Willett WC, Knight EL, Stampfer MJ. Dietary factors and the risk of incident kidney stones in younger women: Nurses' Health Study II. Arch Intern Med. 2004;164(8):885-91.

6. Taylor EN, Stampfer MJ, Curhan GC. Dietary factors and the risk of incident kidney stones in men: new insights after 14 years of follow-up. J Am Soc Nephrol. 2004;15(12):3225-32.

7. Group TRT. Oral vitamin D3 and calcium for secondary prevention of low-trauma fractures in elderly people (Randomised Evaluation of Calcium Or vitamin D, RECORD): a randomised placebo-controlled trial. The Lancet. 2005;365(9471):1621-8.

8. Taylor EN, Stampfer MJ, Curhan GC. Fatty acid intake and incident nephrolithiasis. American journal of kidney diseases : the official journal of the National Kidney Foundation. 2005;45(2):267-74.

9. Taylor EN, Stampfer MJ, Curhan GC. Obesity, weight gain, and the risk of kidney stones. Jama. 2005;293(4):455-62.

10. Lieske JC, de la Vega LSP, Gettman MT, Slezak JM, Bergstralh EJ, Melton ILJ, et al. Diabetes Mellitus and the Risk of Urinary Tract Stones: A Population-Based Case-Control Study. American Journal of Kidney Diseases. 2006;48(6):897-904.

11. Lappe JM, Travers-Gustafson D, Davies KM, Recker RR, Heaney RP. Vitamin D and calcium supplementation reduces cancer risk: results of a randomized trial. The American journal of clinical nutrition. 2007;85(6):1586-91.

12. Taylor EN, Curhan GC. Oxalate intake and the risk for nephrolithiasis. J Am Soc Nephrol. 2007;18(7):2198-204.

13. Jorde R, Sneve M, Figenschau Y, Svartberg J, Waterloo K. Effects of vitamin D supplementation on symptoms of depression in overweight and obese subjects: randomized double blind trial. J Intern Med. 2008;264(6):599-609.

14. Reid IR, Ames R, Mason B, Reid HE, Bacon CJ, Bolland MJ, et al. Randomized controlled trial of calcium supplementation in healthy, nonosteoporotic, older men. Arch Intern Med. 2008;168(20):2276-82.

15. Sneve M, Figenschau Y, Jorde R. Supplementation with cholecalciferol does not result in weight reduction in overweight and obese subjects. European journal of endocrinology. 2008;159(6):675-84.

16. Taylor EN, Curhan GC. Fructose consumption and the risk of kidney stones. Kidney Int. 2008;73(2):207-12.

17. Akoudad S, Szklo M, McAdams MA, Fulop T, Anderson CAM, Coresh J, et al. Correlates of kidney stone disease differ by race in a multi-ethnic middle-aged population: The ARIC study. Preventive medicine. 2010;51(5):416-20.

18. Chang IH, Lee YT, Lee DM, Kim TH, Myung SC, Kim YS, et al. Metabolic syndrome, urine pH, and time-dependent risk of nephrolithiasis in Korean men without hypertension and diabetes. Urology. 2011;78(4):753-8.

19. Wallace RB, Wactawski-Wende J, O'Sullivan MJ, Larson JC, Cochrane B, Gass M, et al. Urinary tract stone occurrence in the Women's Health Initiative (WHI) randomized clinical trial of calcium and vitamin D supplements. The American journal of clinical nutrition. 2011;94(1):270-7.

20. Sorensen MD, Kahn AJ, Reiner AP, Tseng TY, Shikany JM, Wallace RB, et al. Impact of nutritional factors on incident kidney stone formation: a report from the WHI OS. The Journal of urology. 2012;187(5):1645-9.

21. Dai M, Zhao A, Liu A, You L, Wang P. Dietary factors and risk of kidney stone: a case-control study in southern China. Journal of renal nutrition : the official journal of the Council on Renal Nutrition of the National Kidney Foundation. 2013;23(2):e21-8.

22. Ferraro PM, Taylor EN, Gambaro G, Curhan GC. Soda and other beverages and the risk of kidney stones. Clinical journal of the American Society of Nephrology : CJASN. 2013;8(8):1389-95.

23. McAlindon T, LaValley M, Schneider E, Nuite M, Lee JY, Price LL, et al. Effect of vitamin D supplementation on progression of knee pain and cartilage volume loss in patients with symptomatic osteoarthritis: a randomized controlled trial. JAMA. 2013;309(2):155-62.

24. Taylor EN, Curhan GC. Dietary calcium from dairy and nondairy sources, and risk of symptomatic kidney stones. The Journal of urology. 2013;190(4):1255-9.

25. Thomas LD, Elinder CG, Tiselius HG, Wolk A, Akesson A. Ascorbic acid supplements and kidney stone incidence among men: a prospective study. JAMA internal medicine. 2013;173(5):386-8.

26. Ferraro PM, Taylor EN, Gambaro G, Curhan GC. Caffeine intake and the risk of kidney stones. American Journal of Clinical Nutrition. 2014;100(6):1596-603.

27. Oda E. Overweight and high-sensitivity C-reactive protein are weakly associated with kidney stone formation in Japanese men. International Journal of Urology. 2014;21(10):1005-11.

28. Sorensen MD, Chi T, Shara NM, Wang H, Hsi RS, Orchard T, et al. Activity, energy intake, obesity, and the risk of incident kidney stones in postmenopausal women: a report from the Women's Health Initiative. J Am Soc Nephrol. 2014;25(2):362-9.

29. Sorensen MD, Hsi RS, Chi T, Shara N, Wactawski-Wende J, Kahn AJ, et al. Dietary intake of fiber, fruit and vegetables decreases the risk of incident kidney stones in women: A women's health initiative report. Journal of Urology. 2014;192(6):1694-9.

30. Turney BW, Appleby PN, Reynard JM, Noble JG, Key TJ, Allen NE. Diet and risk of kidney stones in the Oxford cohort of the European Prospective Investigation into Cancer and Nutrition (EPIC). European Journal of Epidemiology. 2014;29(5):363-9.

31. Baron JA, Barry EL, Mott LA, Rees JR, Sandler RS, Snover DC, et al. A Trial of Calcium and Vitamin D for the Prevention of Colorectal Adenomas. New England Journal of Medicine. 2015;373(16):1519-30.

32. Ferraro PM, Curhan, G. C., Sorensen, M. D., Gambaro, G., & Taylor, E. N. (2015). ,. Physical Activity, Energy Intake and the Risk of Incident Kidney Stones. The Journal of urology. 2015.

33. Zhao A, Dai M, Chen YJ, Chang HE, Liu AP, Wang PY. Risk factors associated with nephrolithiasis: a case-control study in China. Asia-Pacific journal of public health. 2015;27(2):Np414-24.

34. Ferraro PM, Curhan GC, Gambaro G, Taylor EN. Total, Dietary, and Supplemental Vitamin C Intake and Risk of Incident Kidney Stones. American journal of kidney diseases : the official journal of the National Kidney Foundation. 2016;67(3):400-7.

35. Ferraro PM, Mandel EI, Curhan GC, Gambaro G, Taylor EN. Dietary Protein and Potassium, Diet-Dependent Net Acid Load, and Risk of Incident Kidney Stones. Clinical journal of the American Society of Nephrology : CJASN. 2016;11(10):1834-44.

36. Jorde R, Sollid ST, Svartberg J, Schirmer H, Joakimsen RM, Njolstad I, et al. Vitamin D 20,000 IU per Week for Five Years Does Not Prevent Progression From Prediabetes to Diabetes. The Journal of clinical endocrinology and metabolism. 2016;101(4):1647-55.

37. Yoshimura E, Sawada SS, Lee IM, Gando Y, Kamada M, Matsushita M, et al. Body Mass Index and Kidney Stones: A Cohort Study of Japanese Men. Journal of epidemiology. 2016;26(3):131-6.

38. Ferraro PM, Taylor EN, Gambaro G, Curhan GC. Dietary and Lifestyle Risk Factors Associated with Incident Kidney Stones in Men and Women. The Journal of urology. 2017;198(4):858-63.

39. Ferraro PM, Taylor EN, Gambaro G, Curhan GC. Vitamin D Intake and the Risk of Incident Kidney Stones. The Journal of urology. 2017;197(2):405-10.

40. Lappe J, Watson P, Travers-Gustafson D, Recker R, Garland C, Gorham E, et al. Effect of Vitamin D and Calcium Supplementation on Cancer Incidence in Older Women: a Randomized Clinical Trial. JAMA. 2017;317(12):1234‐43.

41. Leone A, Fernandez-Montero A, de la Fuente-Arrillaga C, Martinez-Gonzalez MA, Bertoli S, Battezzati A, et al. Adherence to the Mediterranean Dietary Pattern and Incidence of Nephrolithiasis in the Seguimiento Universidad de Navarra Follow-up (SUN) Cohort. American journal of kidney diseases : the official journal of the National Kidney Foundation. 2017;70(6):778-86.

42. Shu X, Cai H, Xiang YB, Li H, Lipworth L, Miller NL, et al. Nephrolithiasis Among Middle Aged and Elderly Urban Chinese: A Report from Prospective Cohort Studies in Shanghai. Journal of endourology. 2017;31(12):1327-34.

43. Ferraro PM, Gambaro G, Curhan GC, Taylor EN. Intake of Trace Metals and the Risk of Incident Kidney Stones. Journal of Urology. 2018;199(6):1534-9.

44. Ferraro PM, Taylor EN, Gambaro G, Curhan GC. Vitamin B6 intake and the risk of incident kidney stones. Urolithiasis. 2018;46(3):265-70.

45. Hsi RS, Kabagambe EK, Shu X, Han X, Miller NL, Lipworth L. Race- and Sex-related Differences in Nephrolithiasis Risk Among Blacks and Whites in the Southern Community Cohort Study. Urology. 2018;118:36-42.

46. Kim S, Chang Y, Yun KE, Jung HS, Kim I, Hyun YY, et al. Metabolically healthy and unhealthy obesity phenotypes and risk of renal stone: a cohort study. International Journal of Obesity. 2019;43(4):852-61.

47. Littlejohns TJ, Neal NL, Bradbury KE, Heers H, Allen NE, Turney BW. Fluid Intake and Dietary Factors and the Risk of Incident Kidney Stones in UK Biobank: A Population-based Prospective Cohort Study. European Urology Focus. 2019.

48. Malihi Z, Lawes CMM, Wu Z, Huang Y, Waayer D, Toop L, et al. Monthly high-dose vitamin D supplementation does not increase kidney stone risk or serum calcium: results from a randomized controlled trial. The American journal of clinical nutrition. 2019.

49. Ping H, Lu N, Wang M, Lu J, Liu Y, Qiao L, et al. New-onset metabolic risk factors and the incidence of kidney stones: a prospective cohort study. BJU Int. 2019.

50. Shu X, Cai H, Xiang YB, Li H, Lipworth L, Miller NL, et al. Green tea intake and risk of incident kidney stones: Prospective cohort studies in middle-aged and elderly Chinese individuals. International Journal of Urology. 2019;26(2):241-6.
