# Supplementary material for: Dietary and lifestyle factors for primary prevention of nephrolithiasis: a systematic review and meta-analysis
Source: BMC Nephrol. 2020 Jul 11;21:267. doi: 10.1186/s12882-020-01925-3 (PMC7353736; doi:10.1186/s12882-020-01925-3)
Supplement: Supplementary file 2 — Additional file 2. Summary of Newcastle Ottawa Scale scores for observational studies. [file 12882_2020_1925_MOESM2_ESM.doc]

**Additional file 2.** Summary of Newcastle Ottawa Scale scores for observational studies.

| **Author, year** | **Selection** | **Comparability** | **Outcome** | **Scores** |
| --- | --- | --- | --- | --- |
| Curhan et al, 1997 | 0*** | ** | 0** | 7 |
| Hirvonen et al, 1999 | **** | ** | 0** | 8 |
| Taylor et al, 2004 | 0*** | ** | 0** | 7 |
| Curhan et al, 2004 | 0*** | ** | 0** | 7 |
| Taylor et al, 2005 | 0*** | ** | 0** | 7 |
| Taylor et al, 2005 | 0*** | ** | 0** | 7 |
| Taylor et al, 2007 | 0*** | ** | 0** | 7 |
| Taylor et al, 2008 | 0*** | ** | 0** | 7 |
| Akoudad et al, 2010 | **** | ** | 0** | 8 |
| Chang et al, 2011 | **** | ** | *** | 9 |
| Sorensen et al, 2012 | **** | ** | 0** | 8 |
| Ferraro et al, 2013 | 0*** | ** | 0** | 7 |
| Thomas et al, 2013 | **** | ** | *** | 9 |
| Taylor et al, 2013 | 0*** | ** | 0** | 7 |
| Sorensen et al, 2014 | **** | ** | 0** | 8 |
| Turney et al, 2014 | **** | ** | *** | 9 |
| Sorensen et al, 2014 | **** | ** | 0** | 8 |
| Ferraro et al, 2014 | 0*** | ** | 0** | 7 |
| Oda et al, 2014 | **** | ** | *0* | 8 |
| Ferraro et al, 2015 | 0*** | ** | 0** | 7 |
| Yoshimura et al, 2016 | 0*** | ** | 0** | 7 |
| Ferraro et al, 2016 | 0*** | ** | 0** | 7 |
| Ferraro et al, 2016 | 0*** | ** | 0** | 7 |
| Ferraro et al, 2017 | 0*** | ** | 0** | 7 |
| Ferraro et al, 2017 | 0*** | ** | 0** | 7 |
| Shu et al, 2017 | **0* | ** | 0** | 7 |
| Leone et al, 2017 | 0*** | ** | 0** | 7 |
| Kim et al, 2018 | 0*** | ** | *** | 8 |
| Ferraro et al, 2018 | 0*** | ** | 0** | 7 |
| Ferraro et al, 2018 | 0*** | ** | 0** | 7 |
| His et al, 2018 | **** | ** | *** | 9 |
| Littlejohns et al, 2019 | **** | ** | *** | 9 |
| Shu et al, 2019 | **** | ** | 0** | 8 |
| Ping et al, 2019 | **** | ** | *0* | 8 |
| Krieger et al, 1996^b^ | **** | *0 | 0*0 | 6 |
| Lieske et al, 2006^b^ | **** | ** | **0 | 8 |
| Dai et al, 2013^b^ | 0*0* | ** | 0*0 | 5 |
| Zhao et al, 2015^b^ | 0*0* | ** | 0*0 | 5 |
|  |  |  |  |  |

^a^Newcastle Ottawa Scale uses different criteria for selection, comparability, and outcome to assess the quality of cohort studies and case-control studies. A maximum of 4 stars, 2 stars, and 3 stars can be given to each item of selection, comparability, and outcome respectively.

^b^ Case-control studies. Other studies listed are cohort studies.
