# Supplementary material for: Dietary and lifestyle factors for primary prevention of nephrolithiasis: a systematic review and meta-analysis
Source: BMC Nephrol. 2020 Jul 11;21:267. doi: 10.1186/s12882-020-01925-3 (PMC7353736; doi:10.1186/s12882-020-01925-3)

**Additional file 3.** Risk of bias summary and graph for included randomized controlled trials in meta-analysis.
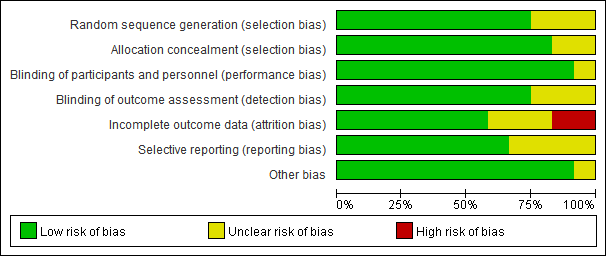


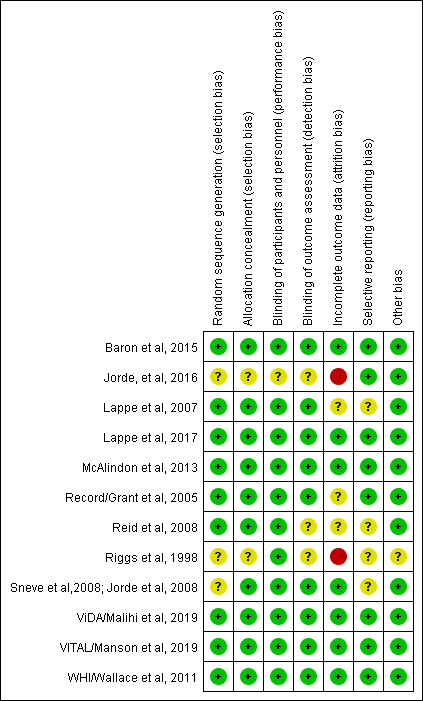

Supplement: Supplementary file 3 — Additional file 3. Risk of bias summary and graph for included randomized controlled trials in meta-analysis. [file 12882_2020_1925_MOESM3_ESM.doc]
