# Supplementary material for: Dietary and lifestyle factors for primary prevention of nephrolithiasis: a systematic review and meta-analysis
Source: BMC Nephrol. 2020 Jul 11;21:267. doi: 10.1186/s12882-020-01925-3 (PMC7353736; doi:10.1186/s12882-020-01925-3)

**Additional file 4.** Sensitivity analyses of the included studies.

1. Sensitivity analysis of studies assessing the association between fruit intake and incident stones.


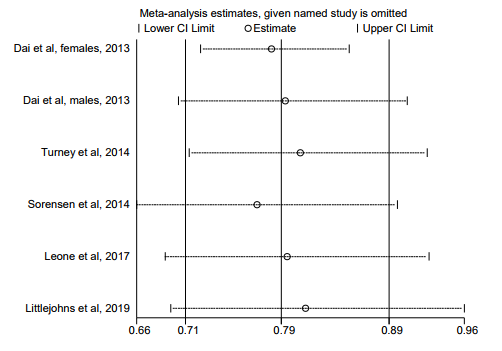


1. Sensitivity analysis of studies assessing the association between vegetables intake and incident stones.


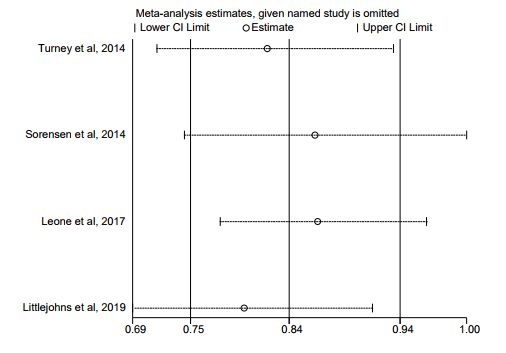


1. Sensitivity analysis of studies assessing the association between fiber intake and incident stones.


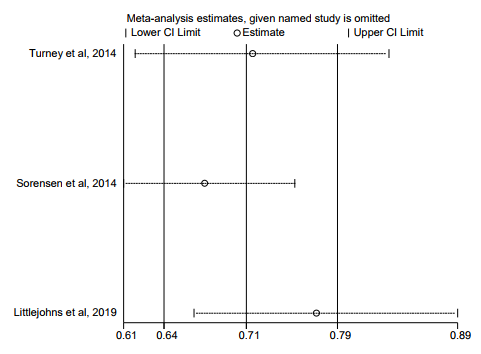


1. Sensitivity analysis of studies assessing the association between meat intake and incident stones.


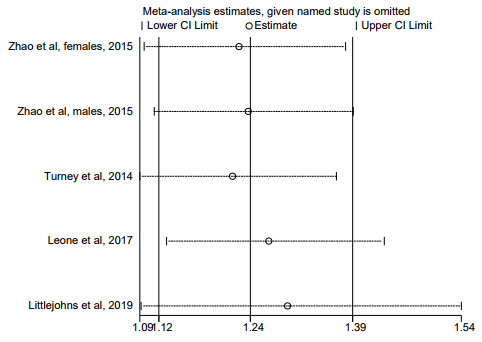


1. Sensitivity analysis of studies assessing the association between animal protein intake and incident stones.


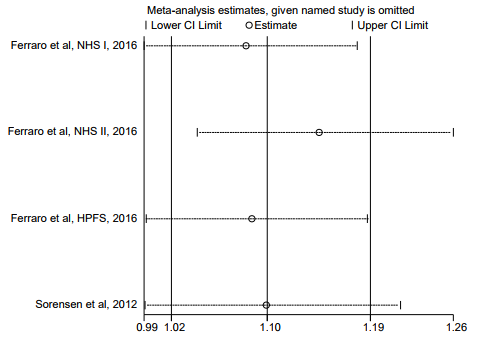


1. Sensitivity analysis of studies assessing the association between spinach and incident stones.


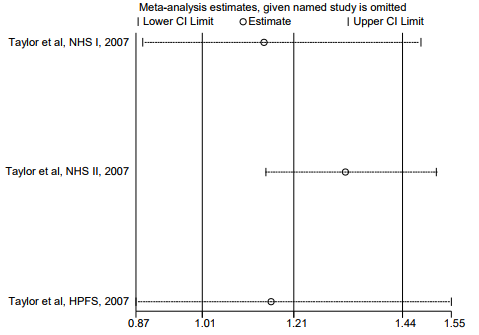


1. Sensitivity analysis of studies assessing the association between oxalate and incident stones.


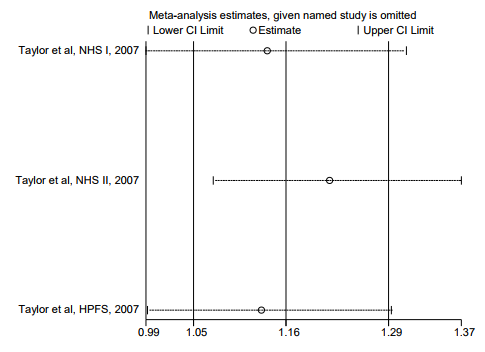


1. Sensitivity analysis of studies assessing the association between DASH style diet and incident stones.


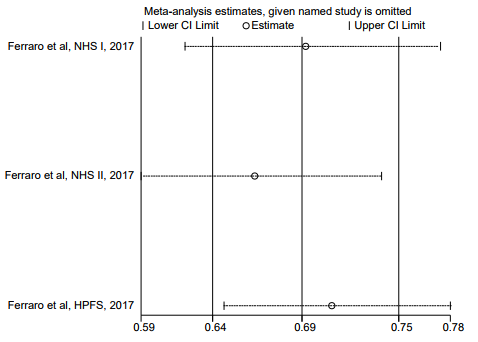


1. Sensitivity analysis of studies assessing the association between dietary magnesium and incident stones.


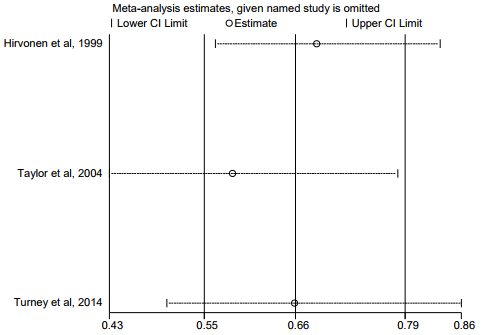


1. Sensitivity analysis of studies assessing the association between dietary sodium and incident stones.


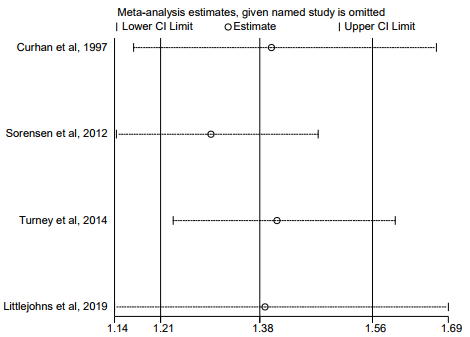


1. Sensitivity analysis of studies assessing the association between dietary potassium and incident stones.


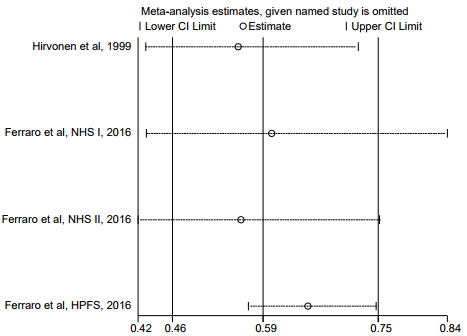


1. Sensitivity analysis of studies assessing the association between dietary calcium and incident stones.


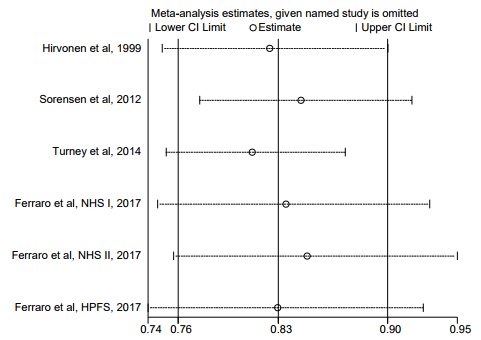


1. Sensitivity analysis of studies assessing the association between total fructose and incident stones.


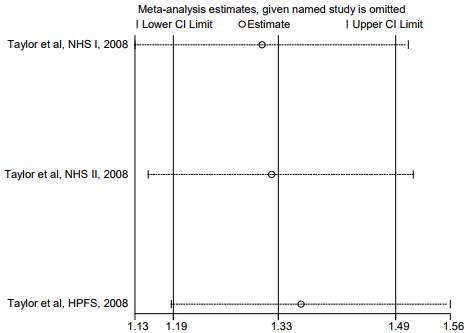


1. Sensitivity analysis of studies assessing the association between caffeine intake and incident stones.


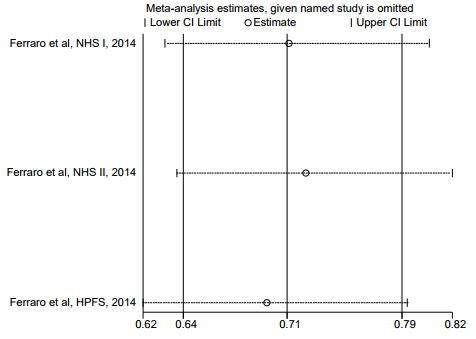


1. Sensitivity analysis of studies assessing the association between fluid intake and incident stones.


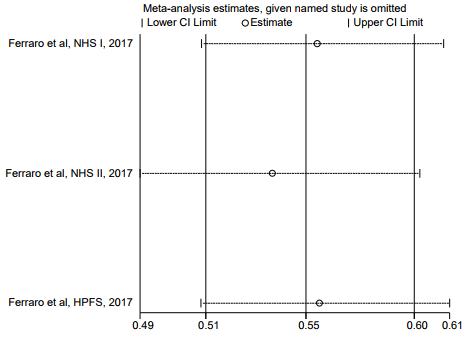


1. Sensitivity analysis of studies assessing the association between tea and incident stones.


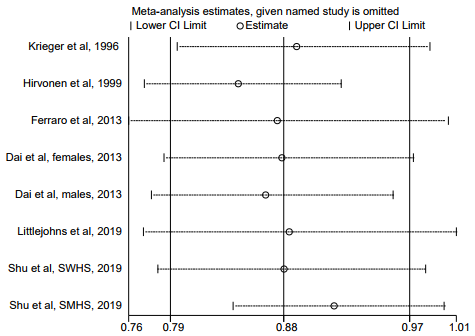


1. Sensitivity analysis of studies assessing the association between coffee and incident stones.


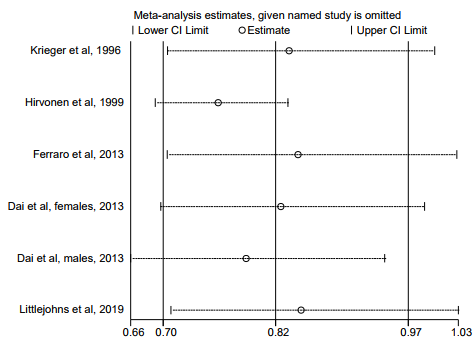


1. Sensitivity analysis of studies assessing the association between water intake and incident stones.


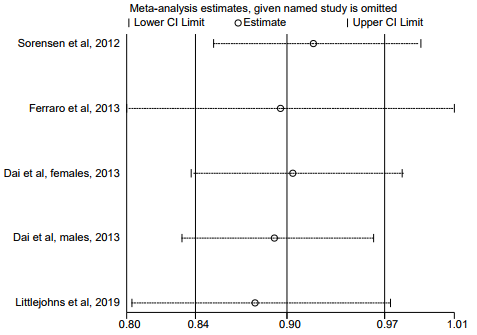


1. Sensitivity analysis of studies assessing the association between beer intake and incident stones.


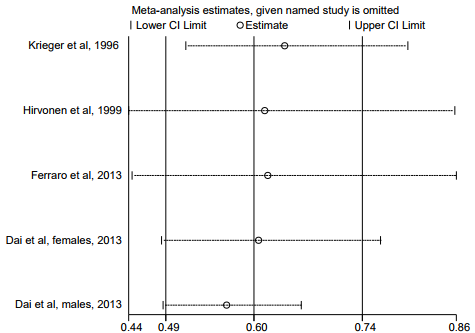


1. Sensitivity analysis of studies assessing the association between alcohol intake and incident stones.


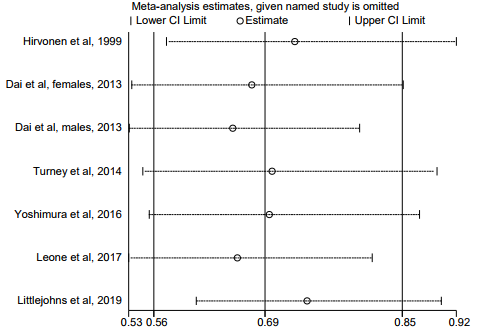


1. Sensitivity analysis of studies assessing the association between soda consumption and incident stones.


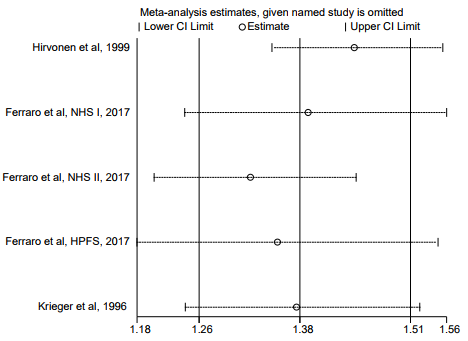


1. Sensitivity analysis of studies assessing the association between total vitamin B6 consumption and incident stones.


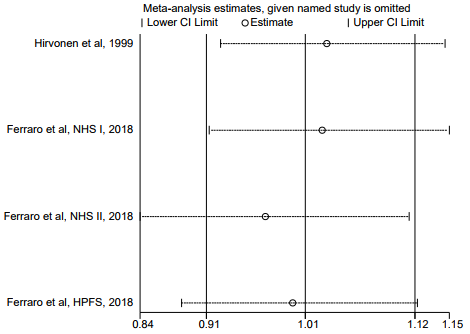


1. Sensitivity analysis of studies assessing the association between total vitamin D consumption and incident stones.


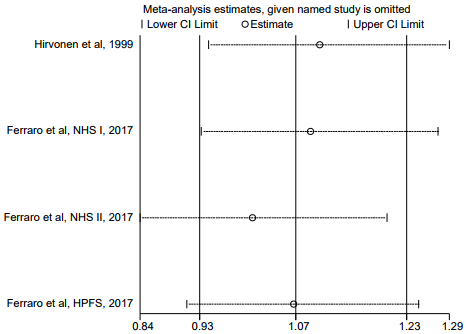


1. Sensitivity analysis of studies assessing the association between total vitamin C consumption and incident stones.


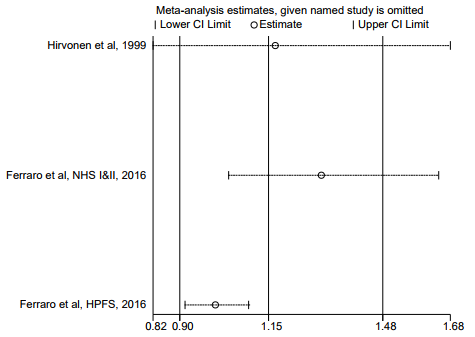


1. Sensitivity analysis of studies assessing the association between supplemental vitamin C consumption and incident stones.


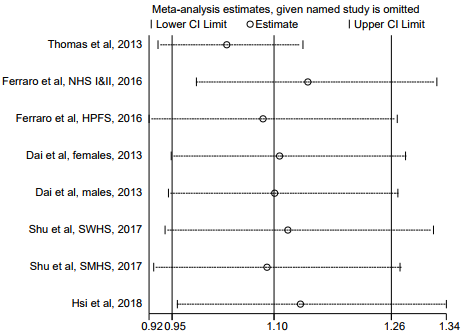


1. Sensitivity analysis of observational studies assessing the association between supplemental vitamin D consumption and incident stones.


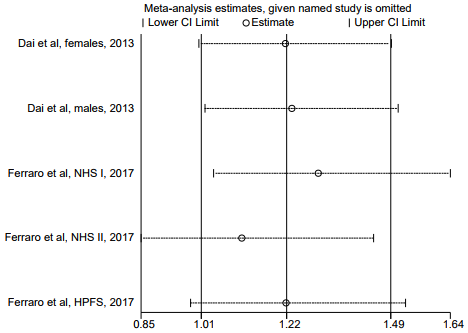


1. Sensitivity analyses of randomized controlled trials ( without and with VITAL) assessing the association between supplemental vitamin D consumption and incident stones.

-Without VITAL study


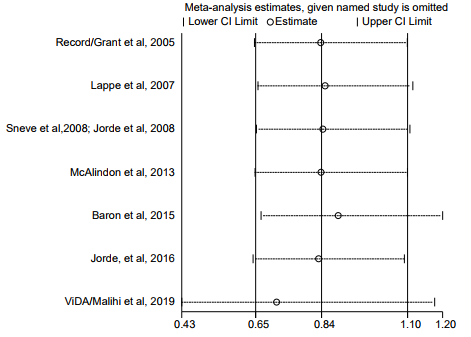


-With VITAL study


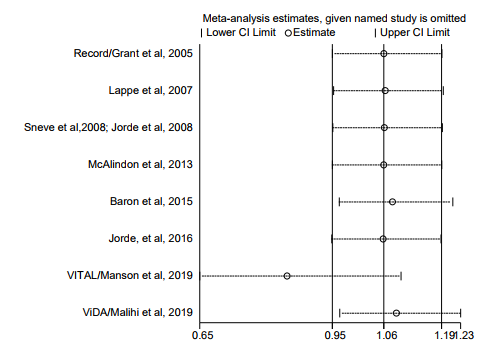


1. Sensitivity analysis of randomized controlled trials assessing the association between calcium supplementation and incident stones.


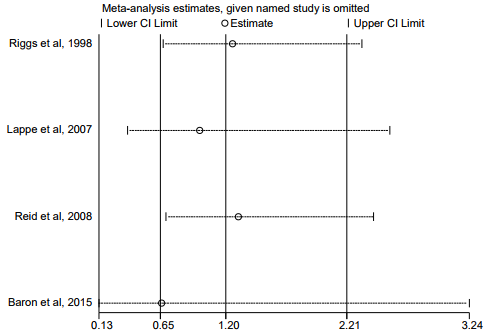


1. Sensitivity analysis of included studies assessing the association between body mass index and incident stones.


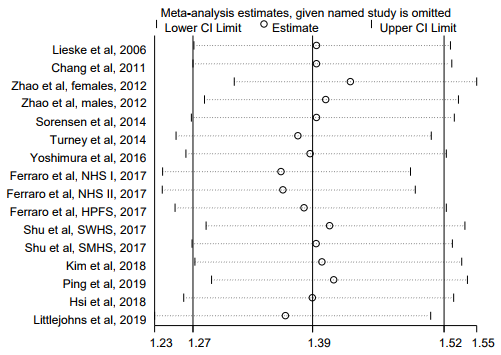


1. Sensitivity analysis of included studies assessing the association between energy intake and incident stones.


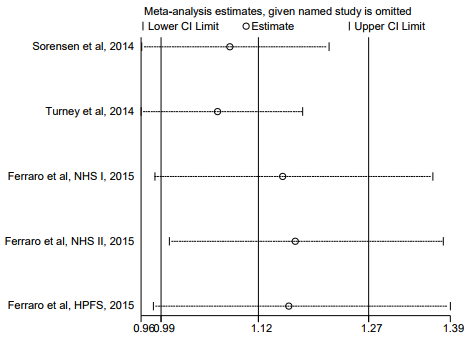


1. Sensitivity analysis of included studies assessing the association between physical activity and incident stones.


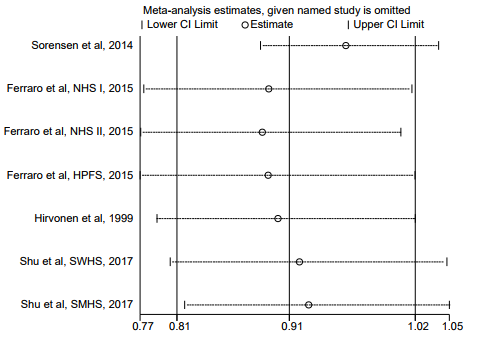

Supplement: Supplementary file 4 — Additional file 4. Sensitivity analyses of the included studies. [file 12882_2020_1925_MOESM4_ESM.doc]
