# Supplementary material for: Dietary and lifestyle factors for primary prevention of nephrolithiasis: a systematic review and meta-analysis
Source: BMC Nephrol. 2020 Jul 11;21:267. doi: 10.1186/s12882-020-01925-3 (PMC7353736; doi:10.1186/s12882-020-01925-3)
Supplement: Supplementary file 5 — Additional file 5. Characteristics of the included studies of incident kidney stones. [file 12882_2020_1925_MOESM5_ESM.doc]

**Additional file 5.** Characteristics of the included studies of incident kidney stones.

| **Author, Year** | **Country** | **Study, Age at baseline, Recruitment period** | **Study design** | **Gender** | **Study period** | **Total no./case** | **Assessment methods** | **Factors assessed** | **Adjusted confounders** |
| --- | --- | --- | --- | --- | --- | --- | --- | --- | --- |
| Curhan et al, 1997 | USA | NHS* I, age 30-55, 1976 | Prospective cohort study | Females | 903849 person-years | 91,731/864 | Food frequency questionnaire; Anthropometric measurements; Self-report of kidney stones | Diet; Vitamins and calcium supplementation | Age, alcohol, BMI*, supplemental calcium, dietary calcium, animal protein, potassium, sucrose, fluid. |
| Hirvonen et al, 1999 | Finland | AlphaTocopherol, Beta-Carotene Lung Cancer Prevention Study, age 50-69years, 1985-1988 | Prospective cohort study | Males | 128170 person-years | 27,001/329 | Validated dietary questionnaire; Self-report of kidney stones | Diet; Beverage; Vitamins and calcium supplementation; physical activity | Age, supplementation group, vocational training, marital status, magnesium, fiber, alcohol |
| Taylor et al, 2004 | USA | HPFS*, age 40-75 years, 1986 | Prospective cohort study | Males | 477700 person-years | 45,619/1,473 | Food frequency questionnaire; Self-report of kidney stones | Diet | Age, BMI, alcohol, thiazide, supplemental calcium, fluid, potassium, sodium, animal protein, phosphorous, magnesium, sucrose, vitamin C, vitamin B6, phytate, vitamin D, dietary calcium |
| Curhan et al, 2004 | USA | NHS II, age 25-42 years, 1989 | Prospective cohort study | Females | 685973 person-years | 96,245/1,223 | Food frequency questionnaire; Self-report of kidney stones | Diet; Vitamins and calcium supplementation | Age, BMI, family history of kidney stones, supplemental calcium, dietary calcium, animal protein, potassium, sodium, sucrose, phytate, fluid |
| Taylor et al, 2005 | USA | NHS I, age 34-59 years, 1976; NHS II, age 27-44 years, 1989; HPFS, age 40-75 years, 1986 | Prospective cohort study | Mixed | NHS I 1980-2000; NHS II 1991-2001; HPFS 1986-2002 | HPFS 45,988/1,609, NHS I 9,3758/1,687, NHS II 101,877/1,531 | Self-report | BMI; Waist circumference; Weight gain | Age, BMI, thiazide, volume intake, alcohol, calcium supplement, dietary calcium, animal protein, potassium, sodium, vitamin C, magnesium |
| Taylor et al, 2005 | USA | NHS I, age 30-55 years, 1976; NHS II, age 25-42 years, 1989; HPFS, age 40-75 years, 1986 | Prospective cohort study | Mixed | NHS I 1986-2000; NHS II 1991-1999; HPFS 1986-2000 | HPFS 46,043/1,479, NHS I 92,079/1,259, NHS II 96,304/1,218 | Food frequency questionnaire; Self-report of kidney stones | Diet | Age, BMI, thiazide, volume intake, alcohol, calcium supplement, dietary calcium, animal protein, potassium, sodium, vitamin C, magnesium |
| Taylor et al, 2007 | USA | NHS I, age 30-55 years, 1976; NHS II, age 25-42 years, 1989; HPFS, age 40-75 years, 1986 | Prospective cohort study | Mixed | NHS I 1984-2002; NHS II 1991-2001; HPFS 1986-2002 | HPFS 45,985/1,627, NHS I 92,872/1,414, NHS II 101,824/1,564 | Food frequency questionnaire; Self-report of kidney stones | Diet | Age, BMI, thiazide, fluid, alcohol, calcium supplement, dietary calcium, animal protein, potassium, sodium, vitamin C, phytate, magnesium |
| Taylor et al, 2008 | USA | NHS I, age 30-55 years, 1976; NHS II, age 25-42 years, 1989; HPFS, age 40-75 years, 1986 | Prospective cohort study | Mixed | NHS I 1980-2002; NHS II 1991-2001; HPFS 1986-2002 | NHS I 93,730/1,711, NHS II 101,824/1,564, HPFS 45,984/1,627 | Food frequency questionnaire; Self-report of kidney stones | Diet | Age, BMI, energy intake, thiazide, fluid, caffeine, alcohol, calcium supplement, percentage of energy from non-fructose carbohydrate and protein, calcium, oxalate, potassium, sodium |
| Akoudad et al, 2010 | USA | Atherosclerosis Risk In Communities study, age 45–64 years, NA | Prospective cohort study | Mixed | Mean 10.8 years | 11,173/90 | Measurements; Self-report and ICD-9 for kidney stones | Waist circumference | Age, gender, race, region, waist circumference, triglycerides, hypertension, diabetes, uric acid, gallstone |
| Chang et al, 2011 | Korea | Hanil General Hospital, Seoul, age 20-59 years, 2002-2003 | Prospective cohort study | Males | Mean (±SD) 5.77±1.32 years | 3,872/118 | Measurements; Kidney ultrasound | BMI | Age, baseline GFR, baseline uric acid levels，incidental hypertension, incidental diabetes |
| Sorensen et al, 2012 | USA | Women’s Health Initiative Observational Study, age 50-79 years, 1993-1998 | Prospective cohort study | Females | 573575 person-years | 78,293/1,952 | Validated food frequency questionnaire; Self-report of kidney stones | Diet; Beverage | Age, race, education, geographic region, calcium supplementation, current estrogen use |
| Ferraro et al, 2013 | USA | NHS I, age 30-55 years, 1976; NHS II, age 25-42 years, 1989; HPFS, age 40-75 years, 1986 | Prospective cohort study | Mixed | NHS I median 13 years; HPFS, NHS II median 8 years | 194,095/4,462 | Validated food frequency questionnaire; Self-report of kidney stones | Beverage | Age, race, region of residence, BMI, furosemide, thiazides, high blood pressure, diabetes, gout, calcium, potassium, phytate, animal protein, vitamin C, total calories, profession (health professionals follow-up study); Mutually adjusted for all the beverages |
| Thomas et al, 2013 | Sweden | Cohort of Swedish Men, age 45-79 years, 1997 | Prospective cohort study | Males | 11 years | 23,355/436 | Self-administered questionnaire; ICD-10 for kidney stones | Vitamins and calcium supplementation | Attained age, education level, BMI, alcohol, dietary calcium, magnesium, potassium, vitamin B6, vitamin C, tea, coffee, smoking status, high blood pressure, diabetes mellitus |
| Taylor et al, 2013 | USA | NHS I, age 30-55 years, 1976; NHS II, age 25-42 years, 1989; HPFS, age 40-75 years, 1986 | Prospective cohort study | Mixed | NHS I 1986-2006 NHS II 1991-2007 HPFS 1986-2006 | HPFS 30,762/1,133, NHS I 94,164 /1,806, NHS II 101,701/2,331 | Food frequency questionnaires; Self-report of kidney stones | Diet | Age, BMI, diabetes, hypertension, thiazide, family history of kidney stones, fluid, alcohol, calcium supplement, dairy calcium, animal protein, potassium, sodium, vitamin C, sodium, oxalate, magnesium, caffeine |
| Sorensen et al, 2014 | USA | Women’s Health Initiative Observational Study, age 50-79 years, 1993-1998 | Prospective cohort study | Females | 610290 person-years | 84,225/2,392 | Semiquantitative food frequency questionnaires; Questionnaire for physical activity; Anthropometric measurement; Self-report of kidney stones | BMI; Physical activity; Energy intake | Age, race, diabetes, calcium supplement, hormone replacement therapy, income, region, water, sodium, animal protein, dietary calcium |
| Turney et al, 2014 | UK | Oxford cohort of the European Prospective Investigation into Cancer and Nutrition (EPIC), aged ≥20 years, 1993-1999 | Prospective cohort study | Mixed | 716105 person-years | 51,336/303 | Food frequency questionnaire; Anthropometric measurements; ICD for kidney stones | Diet; Energy intake; Beverage; BMI | Sex, method of recruitment, region of residence, long-term medical treatment, smoking, alcohol, BMI, diabetes, energy intake |
| Sorensen et al, 2014 | USA | Women’s Health Initiative Observational Study, age 50-79 years, 1993-1998 | Prospective cohort study | Females | 583464 person-years | 80,451/2,390 | Semiquantitative food frequency questionnaire; Self-report of kidney stones | Diet | Age, race, region, diabetes, calcium supplementation, hormone therapy use, BMI, calibrated calorie intake, dietary water, sodium, animal protein, calcium |
| Ferraro et al, 2014 | USA | NHS I, age 30-55 years, 1976; NHS II, age 25-42 years, 1989; HPFS, age 40-75 years, 1986 | Prospective cohort study | Mixed | HPFS median 8.3 years; NHS I median 14 years; NHS II median 8.2 years | NHS I 84,969/1,589, NHS II 90,662/1,812, HPFS 42,252/1,581 | Food frequency questionnaire; Self-report of kidney stones | Beverage | Age, BMI, thiazides, calcium supplements, calcium, phosphate, sodium, potassium, magnesium, fructose, oxalate, phytate, total fluid, alcohol, vitamins B6, C, D |
| Oda et al, 2014 | Japan | Medical Check-up Center, Nagaoka, mean age 51.9 years, 2008-2009 | Retrospective cohort study | Mixed | Mean 3.2 years | Men 1,726/238, Women 992/82 | Questionnaire; Blood tests; Anthropometric measurements; Self-report of kidney stones | BMI; Waist circumference | Age, antihypertensive, antidiabetic or antihyperlipidemic drug use, current smoking, daily alcohol drinking, physical activity, coronary heart disease, stroke |
| Ferraro et al, 2015 | USA | NHS I, age 30-55 years, 1976; NHS II, age 25-42 years, 1989; HPFS, age 40-75 years, 1986 | Prospective cohort study | Mixed | HPFS and NHS I 1986-2006; NHSII 1991-2007 | HPFS 44,964/1,841, NHS II 95,618/2,075, NHS I 74,551/1,439 | Self-report | Physical Activity; Total energy intake | Age, race, BMI, diabetes, calcium supplement, postmenopausal hormone use, geographic region, dietary intake of total energy, calcium, sodium, animal protein, fluids, caffeine, potassium, magnesium, vitamin C, fructose, oxalate, phytate, alcohol, diabetes, high blood pressure, gout, profession, thiazide |
| Yoshimura et al, 2016 | Japan | Cohort study of Japanese men, median age 31 years, 1985 | Prospective cohort study | Males | 19 years | 4,074/258 | Anthropometric measurements; Self-administered questionnaire; Self-report of kidney stones | Beverage; BMI | Age, BMI, systolic blood pressure per 10mmhg, cardiorespiratory fitness, cigarette smoking, alcohol |
| Ferraro et al, 2016 | USA | NHS I, age 30-55 years, 1976; NHS II, age 25-42 years, 1989; HPFS, age 40-75 years, 1986 | Prospective cohort study | Mixed | NHS I median 11.7; NHS II median 11.5 years; HPFS median 11.3 years | NHS I&II 156,735/4,392, HPFS 40,536/1,853 | Food frequency questionnaire; Self-report of kidney stones | Vitamins and calcium supplementation | Age, BMI, thiazide, supplemental calcium, dietary calcium, sodium, potassium, magnesium, fructose, oxalate, phytate, animal protein, total fluid, alcohol |
| Ferraro et al, 2016 | USA | NHS I, age 30-55 years, 1976; NHS II, age 25-42 years, 1989; HPFS, age 40-75 years, 1986 | Prospective cohort study | Mixed | NHS I 1986–2006; NHS II 1991–2011; HPFS 1986–2012 | HPFS 42,919/1,963, NHS I 60,128/1,331, NHS II 90,629/3,014 | Food frequency questionnaire; Self-report of kidney stones | Diet | Age, BMI, diabetes, hypertension, thiazides, supplemental calcium, fluid, sodium, potassium, fructose, oxalate, phytate, alcohol, all other sources of protein |
| Ferraro et al, 2017 | USA | NHS I, age 30-55 years, 1976; NHS II, age 25-42 years, 1989; HPFS, age 40-75 years, 1986 | Prospective cohort study | Mixed | HPFS median 11.5 years; NHS I median 12.1years; NHS II median 11.3 years | 192,126/6,449 | Food frequency questionnaire; Self-report of kidney stones | BMI; Diet; Beverage | Age, race, thiazide, diabetes, hypertension, geographic area, the other risk factors (dash-score was not adjusted for dietary calcium, sugar-sweetened beverages intake because those items were already included in its calculation) |
| Ferraro et al, 2017 | USA | NHS I, age 30-55 years, 1976; NHS II, age 25-42 years, 1989; HPFS, age 40-75 years, 1986 | Prospective cohort study | Mixed | NHS I 1986–2012; NHS II 1991–2011; HPFS 1986–2012 | 193,551/6,576 | Food frequency questionnaire; Self-report of kidney stones | Vitamins and calcium supplementation | Age, region of living, BMI, diabetes, hypertension, thiazides, calcium supplements, calcium, sodium, potassium, magnesium, animal protein, fructose, oxalate, vitamin C, caffeine, alcohol, fluids, dietary vitamin D |
| Shu et al, 2017 | China | Shanghai Women’s Health Study, age 40-70 years, 1996-2000; Shanghai Men’s Health Study, age 40-74 years, 2002-2006 | Prospective cohort study | Mixed | Women 691,836.17 person-years; Men 316,122.06 person-years; Average 8.0 years | Women 67,804/1,451, Men 56,895/1,202 | Validated food frequency questionnaire; Anthropometric measurements; Self-report of kidney stones | BMI; Waist-hip ratio; Physical activity; Vitamins and calcium supplementation | Birth year, pack-year smoking, education, income, energy intake, dietary protein intake, dietary calcium intake, calcium supplement, dietary vitamin C, vitamin C supplement, dietary potassium, dietary magnesium, physical activity, BMI, WHR, CHD/stroke, type 2 diabetes, hypertension, cholelithiasis |
| Leone et al, 2017 | Spain | Seguimiento Universidad de Navarra Follow-up (SUN) Cohort, age 28-46 years, 1999 | Prospective cohort study | Mixed | Mean 9.6 years | 16,094/735 | Validated 136-item food frequency questionnaire; Self-report of nephrolithiasis | Diet; Beverage | Sex, BMI, hypertension, diabetes, marital status, education, number of working hours per week, smoking, physical activity, time spent watching television, total energy intake, total water intake, calcium supplementation, vitamin D supplementation, following a medical nutritional therapy stratified by age, year of recruitment. |
| Kim et al, 2018 | Korea | Kangbuk Samsung Health Study, age ≥18 years, 2002-2015 | Prospective cohort study | Mixed | 1,415,523.0 person-years | 270,190/13,45 | Standardized, self-administered questionnaires; Anthropometric measurements; Abdominal ultrasound; Blood test | BMI | Age, sex, center, year of screening exam, smoking status, alcohol intake, regular exercise, educational level, total cholesterol, HDL, triglycerides, glucose, high-sensitivity c-reactive protein, systolic blood pressure, homeostasis model assessment of insulin resistance |
| Ferraro et al, 2018 | USA | NHS I, age 30-55 years, 1976; NHS II, age 25-42 years, 1989; HPFS, age 40-75 years, 1986 | Prospective cohort study | Mixed | NHS I 1986–2012; NHS II 1991–2011; HPFS 1986–2012 | HPFS 42,919/1,963 NHSI 60,128/1,599 NHSII 90,629/3,014 | Food frequency questionnaire; Self-report of kidney stones | Vitamins and calcium supplementation | Age, BMI, diabetes, hypertension, thiazides, supplemental calcium, fluid, calcium, sodium, potassium, animal protein, fructose, oxalate, vitamin C, caffeine, magnesium, alcohol |
| Ferraro et al, 2018 | USA | NHS I, age 30-55 years, 1976; NHS II, age 25-42 years, 1989; HPFS, age 40-75 years, 1986 | Prospective cohort study | Mixed | NHS I 1986–2012; NHS II 1991–2011; HPFS 1986–2012 | HPFS 42,919/1,963, NHS I 60,003/1,599, NHS II 90,629/3,014 | Food frequency questionnaire; Self-report of kidney stones | Diet | Age, BMI, diabetes, thiazides, supplemental calcium, fluid, salt, animal protein, dietary calcium, potassium, magnesium, fructose, oxalate, total vitamin C, alcohol, caffeine, total vitamin D, supplemental copper, total intakes of zinc, iron, manganese |
| Hsi et al, 2018 | USA | Southern Community Cohort Study, age 40-79 years, 2002-2009 | Prospective cohort study | Mixed | White 116,931 person-years; Black 270,917 person-years | 42,136/1,233 | Self-administered mailed questionnaire or standardized computer-assisted personal interview; Food frequency questionnaire; ICD for incident kidney stones | BMI; Vitamins and calcium supplementation | Race, sex, age as the time scale , education, annual household income, BMI, cardiovascular disease, hypertension, diabetes, high cholesterol, gallstone, total energy intake, daily fluid intake, protein, sugar, fat, calcium, sodium, potassium, magnesium, vitamin C supplementation |
| Littlejohns et al, 2019 | UK | UK Biobank, age 40-69 years, 2006-2010 | Prospective cohort study | Mixed | 2 685 079 person-years | 439,072/2,057 | Touchscreen questionnaires and verbal interviews; Physical measurements; Medical records for kidney stones ascertainment | Diet; Beverage; BMI | Age, sex, Townsend deprivation score, education, ethnicity, smoking, alcohol, BMI, calcium supplementation |
| Shu et al, 2019 | China | Shanghai Women’s Health Study, age 40-70 years, 1996-2000; Shanghai Men’s Health Study, age 40-74 years, 2002-2006 | Prospective cohort study | Mixed | Men 319 211 person-years; Women 696 950 person-years | Men 58,054/1,202, Women 69,166/1,451 | Validated food frequency questionnaire; Anthropometric measurements; Self-report of kidney stones | Beverage | Birth year, pack-year smoking, education, income, physical activity, BMI, WHR, coronary heart disease/stroke, type 2 diabetes, hypertension, cholelithiasis, energy intake, dietary protein intake, dietary calcium intake, calcium supplement, dietary vitamin C intake, vitamin C supplement, dietary potassium, dietary magnesium, dietary oxalate |
| Ping et al, 2019 | China | Qingdao Port Cardiovascular Health Study, age ≥18 years, 2000 | Prospective cohort study | Mixed | Mean 33.5 months | 9,667/676 | Standardized questionnaire; Anthropometric measurements; Abdominal ultrasound | BMI | Age, marital status, household income, cigarette smoking, alcohol drinking, hypertension, diabetes, obesity, total cholesterol, triglycerides, low-density lipoprotein cholesterol, high-density lipoprotein cholesterol, creatinine, uric acid |
| Krieger et al, 1996 | USA | Group Health Cooperative, Puget Sound region, age 25-55 years, 1989-1992 | Case control study | Males | NA | 632/240 | Standardized telephone interview | Beverage | Age, low-fat or weight reduction diet, beer drinking, antacid use, diuretic, demographic characteristics, family history of urolithiasis |
| Lieske et al, 2006 | USA | Medical Record Review Case Control Study, mean age 43.8 years,1980-1999 | Case control study | Mixed | NA | 520/260 | Medical record review | BMI; Diet | Age, thiazide use, diabetes, hypertension, BMI |
| Zhao et al, 2015 | China | Health Management Center, Southern China, age ≥18years, 2010-2011 | Case control study | Mixed | NA | Men 458/232; Women 820/493 | Interviewer-administered questionnaire ; Measurements; Blood tests | BMI; Waist hip ratio; Diet | Age, education, family history of kidney stones |
| Dai et al, 2013 | China | Health Management Center, Southern China, age ≥18years, 2010-2011 | Case control study | Mixed | NA | 2,006/1,019 | Food frequency questionnaire; Measurements | Diet; Beverage; vitamin and calcium supplementation | Age, BMI, smoke status, occupation, education, history of hypertension |

|  | **Country** | **Population** | **Study design** | **Gender** | **Total no./case** | **Intervention** | **Control** | **Follow-up** | **Primary outcome** |
| --- | --- | --- | --- | --- | --- | --- | --- | --- | --- |
| Riggs et al, 1998 | USA | Community-dwelling postmenopausal women , age 61-70 years | Randomized controlled trial | Females | 236/1 | 1,600 mg Ca/d | Placebo | 4 years | Serum Parathyroid Hormone Level, Bone Turnover, and Bone Loss |
| RECORD/Grant et al, 2005 | UK | Adults with a history of low-trauma osteoporotic fracture, age≥70 years | Randomized controlled trial | Mixed | 5,292/4 | 800 IU Vit D3/d; 1,000 mg Ca/d;  800 IU Vit D3/d + 1,000 mg Ca/d | Placebo | median 3.8 years | Incidence of new low-energy fractures |
| Lappe et al, 2007 | USA | Community-dwelling postmenopausal women without cancers at entry, age≥55 years | Randomized controlled trial | Females | 891/4 | 1,400–1,500 mg Ca/d + 1,100 IU Vit D3/d; 1,400–1,500 mg Ca/d | Placebo | 4 years | Skeletal status and calcium economy |
| Sneve et al,2008; Jorde et al, 2008 | Norway | Adults with BMI 28-47kg/m2 , age 21-75 years | Randomized controlled trial | Mixed | 445/2 | 40,000 IU Vit D3/wk + 500 mg Ca/d;  20,000 IU Vit D3/wk + 500 mg Ca/d | 2 placebos/wk + 500 mg Ca/d | 1 years | Symptoms of depression |
| Reid et al, 2008 | New Zeland | Healthy non-osteoporotic men, mean age 56 years | Randomized controlled trial | Males | 290/1 | 600 and 1,200 mg Ca/d | Placebo | 2 years | Bone mineral density |
| WHI/Wallace et al, 2011 | USA | Postmenopausal women, age 50–79 years | Randomized controlled trial | Females | 36,282/830 | 400 IU Vit D3/d + 1,000 mg Ca/d | Placebo | 7 years | Incidence of fracture |
| McAlindon et al, 2013 | USA | Patients with knee osteoarthritis, mean age 62.4 years | Randomized controlled trial | Mixed | 146/2 | 2,000 IU Vit D3/d | Placebo | 2 years | Knee pain and cartilage volume |
| Baron et al, 2015 | USA | Patients with recently diagnosed adenomas , mean age 57 years | Randomized controlled trial | Mixed | 2,259/47 | 1,000 IU Vit D3/d;  1,000 Vit D3/d + 1,200 mg Ca/d;  1,200 mg Ca/d | Placebo | 3 years | Incidence of colonic adenoma |
| Jorde, et al, 2016 | Norway | Adults with prediabetes, mean age 62 years | Randomized controlled trial | Mixed | 501/3 | 20,000 IU Vit D3/wk | Placebo | 5 years | Progression to Type2 diabetes |
| Lappe et al, 2017 | USA | Healthy postmenopausal women, mean age 65 years | Randomized controlled trial | Females | 2,197/26 | 2,000 IU/d Vit D3+ 1,500 mg Ca/d | Placebo | 4 years | Incidence of all-type cancer (excluding nonmelanoma skin cancers) |
| ViDA/Malihi et al, 2019 | New Zeland | Adults, age 50–84 years | Randomized controlled trial | Mixed | 5,108/158 | 100,000 IU Vit D3/month | Placebo | median 3.3 years | Incident cardiovascular disease and death |

*Abbreviations: BMI, body mass index. NHS I, Nurses’ Health Study I. NHS II, Nurses’ Health Study II. HPFS, Health Professional Follow-up Study. HRT, hormone replacement therapy. RASS-inhibitors, renin-angiotensin-aldosterone system inhibitors. RECORD, Randomized Evaluation of Calcium or Vitamin D. WHI, Women Health Initiative. VITAL, Vitamin D and Omega-3 Trial. ViDA, Vitamin D Assessment. Ca, calcium. Vit D3, vitamin D3.
