# Supplementary material for: Dietary and lifestyle factors for primary prevention of nephrolithiasis: a systematic review and meta-analysis
Source: BMC Nephrol. 2020 Jul 11;21:267. doi: 10.1186/s12882-020-01925-3 (PMC7353736; doi:10.1186/s12882-020-01925-3)
Supplement: Supplementary file 6 — Additional file 6. Funnel plot (A) and Egger’s test (B) for publication bias and small study effects among studies assessing the association between body mass index and risk of incident nephrolithiasis. [file 12882_2020_1925_MOESM6_ESM.docx]

**Additional file 6.** Funnel plot (A) and Egger’s test (B) for publication bias and small study effects among studies assessing association between body mass index and risk of incident nephrolithiasis.


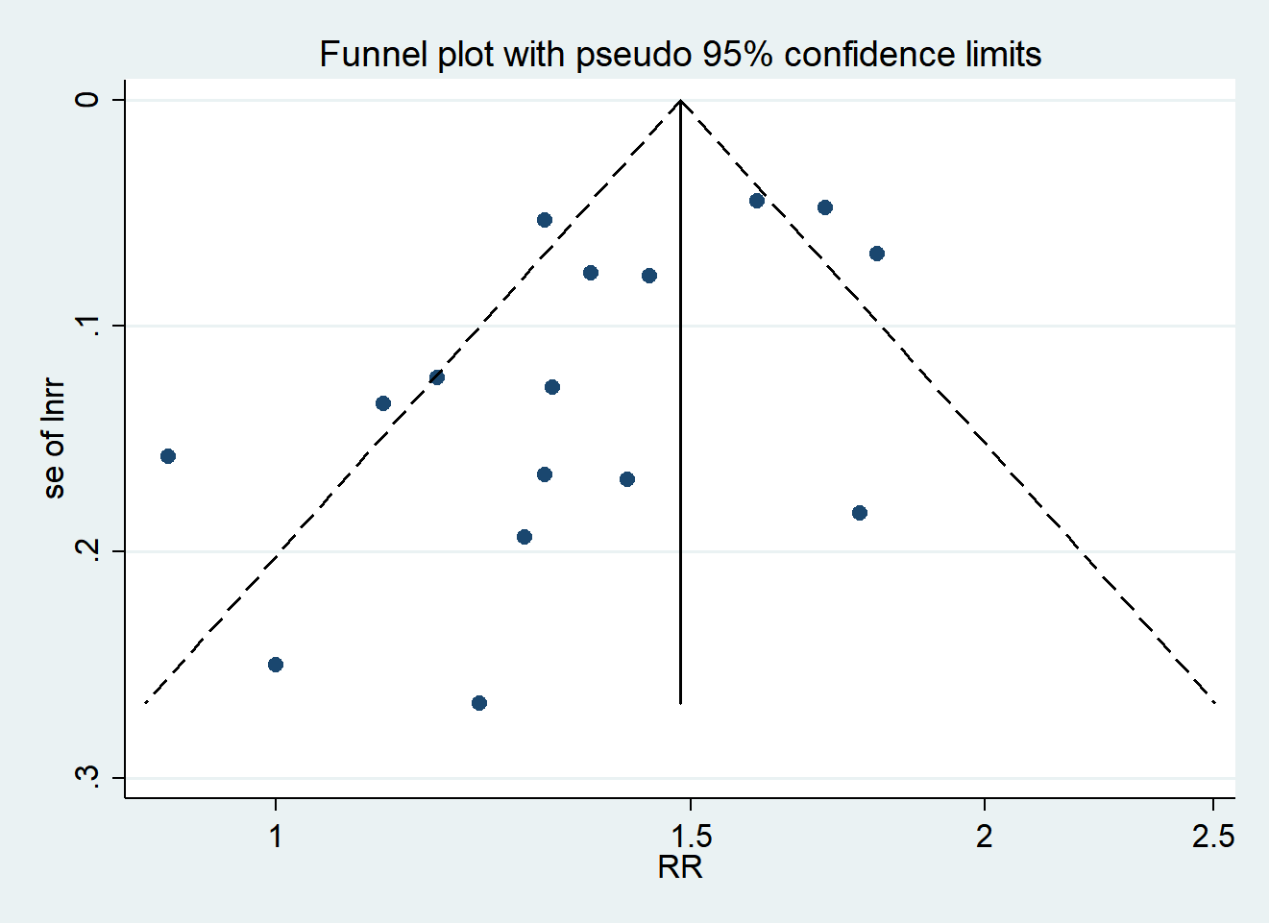


A

The figure is characterized with circles representing included studies, the solid line and dashed lines indicating the pooled estimate and pseudo-95% confidence limits respectively. The result showed several dots fell outside the interval and obvious asymmetry on visual inspection.


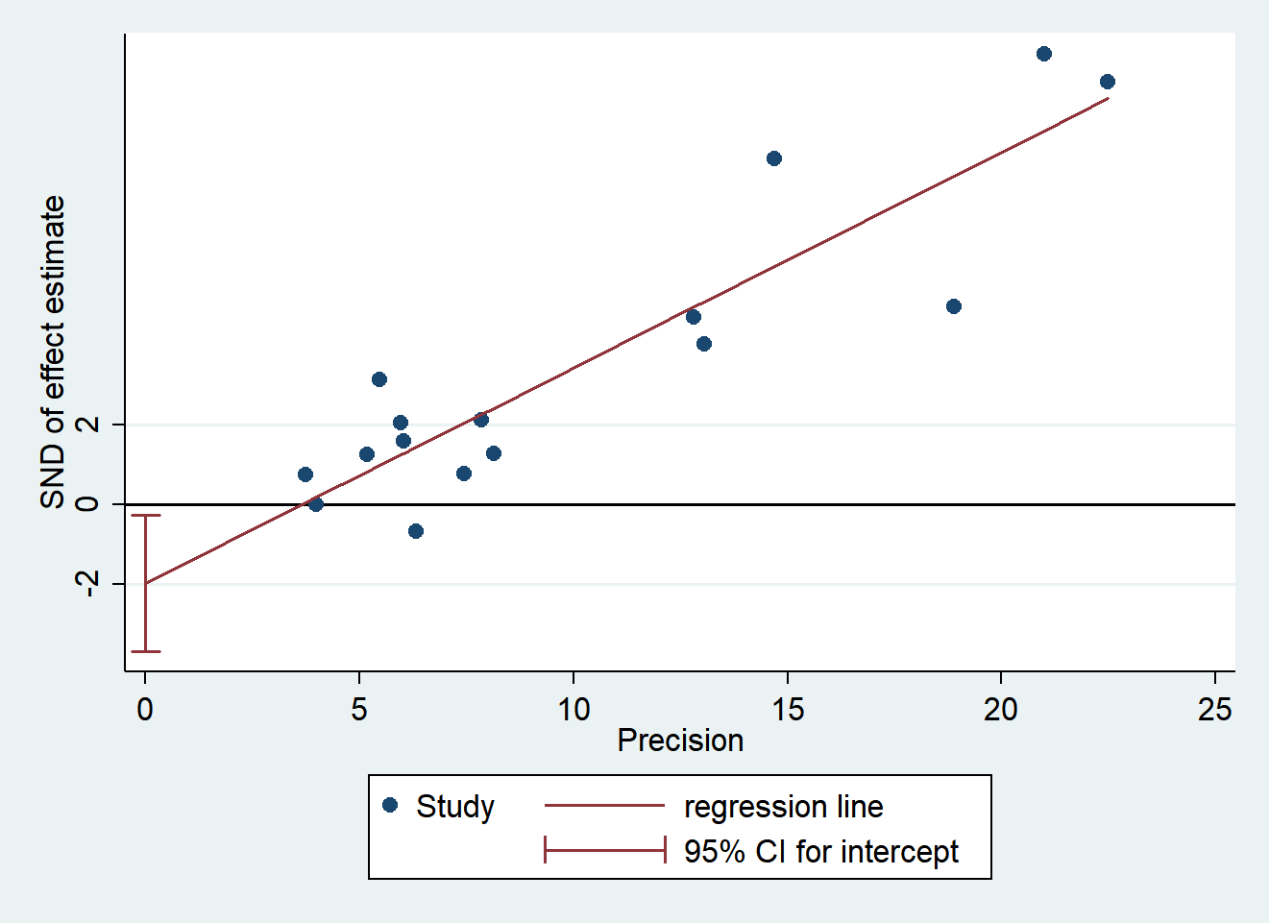


B

Egger’s test yielded P= 0.026, indicating significant small study effects.
