# Supplementary material for: Dietary and lifestyle factors for primary prevention of nephrolithiasis: a systematic review and meta-analysis
Source: BMC Nephrol. 2020 Jul 11;21:267. doi: 10.1186/s12882-020-01925-3 (PMC7353736; doi:10.1186/s12882-020-01925-3)
Supplement: Supplementary file 7 — Additional file 7. Subgroup meta-analyses and meta-regression of body mass index and incident kidney stones. [file 12882_2020_1925_MOESM7_ESM.doc]

**Additional file 7.** Subgroup meta-analyses and meta-regression of body mass index and incident kidney stones.*****

|  | **n** | **RR (95%CI)** | **I^2^ ,%** | **P^a^** | **P^b^** |
| --- | --- | --- | --- | --- | --- |
| All studies | 16 | 1.39 (1.27-1.52) | 71.2 | < 0.001 |  |
| Gender |  |  |  |  | 0.888 |
| Males | 5 | 1.37 (1.22 -1.54) | 0.0 | 0.688 |  |
| Females | 5 | 1.38 (1.12 -1.70) | 85.3 | < 0.001 |  |
| Mixed | 6 | 1.39 (1.23 -1.58) | 67.3 | 0.009 |  |
| Geographic region |  |  |  |  | 0.012 |
| Europe | 2 | 1.61 (1.48 -1.75) | 0.0 | 0.592 |  |
| Americas | 6 | 1.53 (1.36 -1.72) | 66.1 | 0.012 |  |
| Asia | 8 | 1.23 (1.13 -1.34) | 3.2 | 0.405 |  |
| Number of cases |  |  |  |  | 0.075 |
| <1000 cases | 9 | 1.21 (1.02 -1.44) | 74.8 | < 0.001 |  |
| ≥ 1000 cases | 7 | 1.47 (1.33 -1.61) | 38.3 | 0.137 |  |
| Study design |  |  |  |  | 0.024 |
| Case control studies | 3 | 0.98 (0.78 -1.24) | 0.0 | 0.615 |  |
| Cohort studies | 13 | 1.44 (1.32 -1.57) | 69.0 | < 0.001 |  |
| Study quality |  |  |  |  | 0.021 |
| NOS ≤ 6 | 2 | 0.93 (0.71 -1.21) | 0.0 | 0.722 |  |
| NOS > 6 | 14 | 1.44 (1.32 -1.56) | 66.9 | < 0.001 |  |
| Assessment methods |  |  |  |  | 0.043 |
| Self-report | 4 | 1.58 (1.39 -1.79) | 73.1 | 0.011 |  |
| Measurement | 12 | 1.29 (1.15 -1.44) | 61.0 | 0.003 |  |
| Study period^c^ |  |  |  |  | 0.021 |
| < 10 years | 8 | 1.33 (1.21 -1.48) | 58.6 | 0.018 |  |
| ≥ 10 years | 5 | 1.65 (1.50 -1.81) | 35.3 | 0.186 |  |
| Adjustment for confounders |  |  |  |  |  |
| Age |  |  |  |  | 0.268 |
| Yes | 13 | 1.33 (1.19 -1.50) | 74.2 | < 0.001 |  |
| No | 3 | 1.53 (1.34 -1.74) | 49.2 | 0.139 |  |
| Smoking |  |  |  |  | 0.134 |
| Yes | 5 | 1.27 (1.17 -1.38) | 0.0 | 0.727 |  |
| No | 11 | 1.46 (1.31 -1.61) | 69.0 | < 0.001 |  |
| Alcohol |  |  |  |  | 0.113 |
| Yes | 7 | 1.50 (1.31 -1.71) | 78.4 | < 0.001 |  |
| No | 9 | 1.27 (1.11 -1.46) | 63.0 | 0.006 |  |
| Hypertension |  |  |  |  | 0.678 |
| Yes | 10 | 1.40 (1.25 -1.58) | 73.2 | < 0.001 |  |
| No | 6 | 1.34 (1.12 -1.59) | 73.0 | 0.002 |  |
| Type 2 diabetes |  |  |  |  | 0.372 |
| Yes | 10 | 1.44 (1.27 -1.62) | 66.1 | 0.002 |  |
| No | 6 | 1.31 (1.13 -1.52) | 75.7 | 0.001 |  |
| Fluid |  |  |  |  | 0.042 |
| Yes | 4 | 1.60 (1.41 -1.80) | 64.8 | 0.036 |  |
| No | 12 | 1.30 (1.17 -1.45) | 60.9 | 0.003 |  |
| Diet |  |  |  |  | 0.206 |
| Yes | 6 | 1.49 (1.31 -1.70) | 71.1 | 0.004 |  |
| No | 10 | 1.31 (1.17 -1.48) | 65.2 | 0.002 |  |
| Physical activity |  |  |  |  | 0.247 |
| Yes | 7 | 1.46 (1.29 -1.66) | 77.9 | < 0.001 |  |
| No | 9 | 1.30 (1.12 -1.50) | 66.0 | 0.003 |  |
| Thiazide |  |  |  |  | 0.027 |
| Yes | 4 | 1.63 (1.46 -1.83) | 53.5 | 0.092 |  |
| No | 12 | 1.31 (1.18 -1.44) | 60.8 | 0.003 |  |

*Incident kidney stones are defined as the first kidney stones diagnosed. n denotes the number of comparisons. I^2^ means heterogeneity estimate. RR, relative risk. CI, confidence interval. NOS, Newcastle-Ottawa Scale.

^a^P values for heterogeneity within subgroups.

^b^P values for heterogeneity between subgroups.

^C^Only cohort studies were included for analysis.
